# Ethnic and religious identities in Russian penal institutions: A case study of Uzbek Transnational Muslim prisoners

**DOI:** 10.12688/openreseurope.16142.2

**Published:** 2024-01-09

**Authors:** Rustamjon Urinboyev, Judith Pallot

**Affiliations:** 1 Aleksanteri Institute, University of Helsinki, Helsinki, Uusimaa, FI-00014, Finland; 2 Sociology of Law, Lund University, Lund, Scania, Box 42, S-221 00 Lund, Sweden; 3 Department of Social Sciences and Humanities, Tashkent State University of Economics, Tashkent, Uzbekistan

**Keywords:** Russia; prisons; penality; Muslim prisoners; Uzbeks; ethnicity; Islam; radicalisation.

## Abstract

Russia has become one of the main migration hubs worldwide following the collapse of the Soviet Union. The vast majority of migrant workers travel to Russia from three Central Asian countries. However, Russian immigration laws and policies are ambiguous and highly punitive. The result is that many migrants resort to undocumented status working in the shadow economy, which places them in a disadvantaged and precarious position. In this position they are vulnerable to becoming targets of the Russian criminal justice system as they take to crime to overcome economic uncertainty, become embroiled in interpersonal conflicts ending in violence, or fall victim to fabricated criminal charges initiated by Russian police officers under pressure to produce their monthly quota of arrests. The impact on Russian penal institutions is that they have become ethnically, culturally, and religiously diverse sites as a consequence of the incarceration of growing numbers of transnational prisoners. Using person-to-person interviews conducted in Uzbekistan with men and women who served sentences in Russian penal institutions during the past two decades, we show in this article how the large-scale migratory processes have transformed Russian prisons into sites of ethnic and religious plurality, in which formal rules and informal sub-cultures - the colony regime, so-called thieves' law ( *vorovskoy zakon*), ethnic solidarity norms, and Sharia law - coexist and clash in new ways compared with the status quo ante. Thus, we argue there is a need to revise the prevailing understanding about the power dynamics in Russian penal institutions. Our findings undermine the prison service's insistence of the ethnic and ethno-religious neutrality and 'cosmopolitanism' of Russian penal space, which is presented as a latter-day manifestation of the Soviet-era 'friendship of nations' policy. Russian prisons today must be understood as sites of ethnic and religious pluralism.

## Plain Language summary

This article addresses the question of the experiences of Muslim transnational prisoners in the Russian Federation. It is based on interviews the project GULAGECHOES conducted in Uzbekistan with men and women who previously had served sentences of varying lengths and severity in Russian penal facilities over the past two decades. Unlike in Soviet times when Uzbeks were counted as Russian nationals, today they are legally defined as ‘foreigners’, and their presence in large numbers in the country’s prisons makes the situation in Russia more like in Europe, which has high numbers of prisoners from abroad. The focus of our investigation is on group formation among Muslim prisoners and their relationship between the different power hierarchies that already exist in Russian penitentiaries. We do this through discussing the journey of one Uzbek in-migrant to Russia, through prison and back to Uzbekistan. Our aim is to contest some of the stereotypical assumptions about the linkage between migrants, the traditional prisoner sub-culture of the thieves-in-law, Islamic belief and practices and violent radicalisation. We show that the social interactions among prisoners and the motives lying behind them are multi-faceted and complex. After the introduction and sections giving the contextual information needed to understand the Russian prison system, we discuss the experiences of Muslim transnational prisoners under headings dealing with Islam and prisoner hierarchies; surveillance by prison staff; ethnic solidarity; and contact with the outside world. We conclude that the past two decades have seen the emergence of a far more complicated and plural power geometry between the prison administration and prisoners and between Russian traditional criminal sub-culture and transnational Muslim prisoners.

## Introduction

Russia has become one of the main migration hubs globally following the collapse of the Soviet Union. ^1^ The majority of migrant workers originate from the three Central Asian states of Uzbekistan, Tajikistan, and Kyrgyzstan. According to 2022 statistics, there were about 1.45 million Uzbek, nearly one million Tajik, and 562 thousand Kyrgyz nationals in Russia. ^2^ The average Central Asian migrant is a young male with secondary education and a poor command of the Russian language, originating from the rural areas or small towns of Central Asia where unemployment rates remain exceptionally high. ^3^ Central Asian migrants primarily work in construction, trade, transportation, service, agriculture, housing, and communal services. Although Central Asian migrants enter Russia legally thanks to the visa-free regime under a Commonwealth of Independent States (CIS) agreement, many migrants become ‘undocumented’ after failing to obtain a work permit and residence registration. ^4^ This is a product of the highly restrictive and inconsistent Russian immigration laws, which make it difficult for Central Asian migrants to be ‘fully legal,’ or documented, in Russia. ^5^ The millions of Central Asian migrants who have undocumented status are compelled to work in the shadow economy, where they can survive without immigration documents and language skills. ^6^ However, the shadow economy employment often puts migrants in a disadvantaged and precarious position, forcing them to pay bribes to Russian police officers and leaving them vulnerable to fraud and deceit by employers and intermediaries. ^7^ As the migrants take to crime to overcome their economic uncertainty, they make recourse to violence in interpersonal conflicts, or fall victim to fabricated criminal charges initiated by Russian police officers under pressure to produce their monthly quota of arrests. Consequently, a constant sense of insecurity associated with a fear of discrimination, injustice, exploitation, abuse, and illegality are the feature of many Central Asian migrants’ everyday lives. ^8^ These are common consequences of the exclusionary process labelled in recent western scholarship as ‘crimmigration’, to describe the intersection of criminal law and immigration law, which invariably culminates in the expulsion of the migrants caught in its vice. ^9^

The crimmigration literature primarily focuses upon the waves of vulnerable and poor migrants who arrived in western Europe in the aftermath of the WWII from the global south. For the radical American sociologist Loic Waquant, the increase in the number of people held in various forms of detention in western Europe associated with this migration can be equated with the over-representation of poor blacks detained in US prisons. The immigrants to Europe, he asserts, are the ‘blacks of American jails’, and their detention the ‘confinement of disproportionality’, This, he arguyes, is part of the broader process of the criminalisation of poverty in the developed industrialized countries. ^10^ While the analogy draws attention to the broader process of the criminalization of poverty in the developed countries, it is does not capture the differences in the experiences between migrants detained on criminal and administrative charges, which are important in determining the ultimate fate of migrants. 

There have been few studies of the experiences of foreigners in prison. Those that do exist have shown that the imprisonment has led to a bifurcation in the treatment of prisoners in western prisons, regardless of their legal status. Uglevik and Damsa believe that, as a group, trasnational prisoners challenge the inclusivity goal of the modern European prison, ‘nordic exceptionalism’ included. ^11^ In principle, prison services can be tailored to meet transnational prisoners’ needs, but this is unusual. Typically, they do not benefit from resocialisation interventions designed for nationals, because of the assumption that they will be deported as soon as their sentence has expired. 

The successive waves of post-war migrations into Europe from the global south is credited with changing the ethnic and cultural composition of prisoner populations in Europe over a relatively short period and it coincided with the so-called ‘punitive turn’ that saw prison populations soar from the 1970s. ^12^ In the USSR, the prison story unfolded differently. Mass internal labour relocations during the communist period were central to economic development of resource-rich frontiers in the country, which was achieved by centralised planning and regulation. Forced penal labour was an integral part of the process. Throughout the Soviet period, the prison service was a unitary agency directed from Moscow, with universally applicable rules across the country, and Russian was its official language. Prisoners were moved across cultural and ethnic boundaries according to the capacity of the penal estate, regional demand for labour and prisoner characteristics, and all prisoners were subjected to equally degrading and inhuman conditions. As borders were largely closed to the outside world, transnational, or foreign, prisoners were rare. This changed in 1991, when domestic inter-regional migrations between the former Soviet Socialist Republics were transformed overnight into international, trans border flows between sovereign states. 

In the late Soviet era, the small number of foreign nationals convicted of offences in the USSR were held in a special facility for foreign prisoners in the settlement of Leplei, in the Mordoviyan ASSR. Today, there are few countries in the global north that segregate foreign nationals. In Europe, only the UK and Norway have separate ‘specialist facilities’ for foreign nationals. These are justified on the grounds that services could be improved by segregation to meet non-nationals’ specific needs than when the latter are spread through the prison estate. In the UK, for example, Bullwood Hall and Canterbury prison, are specialist facilities which aim to overcome the language and cultural barriers foreign prisoners suffer. But the policy is controversial, as segregation can form the basis for discriminatory treatment on grounds of citizenship. Following Council of Europe Committee for the Prevention of Torture recommendations, Russia partially desegregated the facility for foreign nationals in correctional colony no. 22 in Leplei in 2010 by placing Russian nationals in the facility, although foreign prisoners, primarily from the ‘far abroad’ still are the majority. ^13^

In this article, our interest is in the transnational migrants from the ‘near abroad’ who are subject to criminal sanction, leading to a prison sentence served in the Russian Federation and followed by deportation. They include both those migrants who prior to arrest had legal status in Russia and those who are the variously named ‘undocumented’, ‘illegal’, or ‘informal’ migrants. The offences for which they are convicted cover a spectrum from violations of migration laws, the number of which subject to criminal sanction has increased since the turn of the century, and others ranging from the relatively minor offenses, to serious crimes against the person and property. The placement of the citizens of the former Soviet republics has not changed since the end of the USSR in that the majority, as before 1991, are detained in regular correctional colonies together with Russian citizens. However, the similarity with the situation ante is illusory as the migrants, now classified as ‘foreigners’ or ‘aliens’ , have specific rights in the Russian Federation criminal correction code and are treated differently compared with Russian nationals. ^14^ Culturally, linguistically and politically, the distance between these prisoners and Russian nationals has increased as the last vestiges of a common Soviet identity have been erased. Whereas in the past, there was shared penal experience for people in the USSR, the newly re-classified foreign prisoners today find themselves in a socio-legal environment with unfamiliar rules of conduct and social behaviour, and this is especially true of Central Asian nationals. Meanwhile, the responses in terms of the self- and group-identification of the transnational prisoners have been affected by the changing perception in Russia of the risk different migrant groups pose to the social, political and demographic security of the Russian Federation. Three decades after the USSR’s collapse, Russia has followed west European countries in using the criminal-justice system to control immigrant numbers, in the process reinforcing their outsider status.

## Quantifying Transnational Prisoners in the Russian Federation

After a brief period in the 1990s, the Russian Prison Service or Federal Service for the Execution of Punishments, the FSIN ( *Federal’naya Sluzhba Ispolneniya Nakazaniya*), returned to a culture of secrecy and lack of transparency typical of the Soviet era. As a consequence, there is an absence of detailed and verifiable statistics about the number of foreign migrant workers serving sentences in Russian correctional institutions ( *ispravitel’nye kolonii*) and detained in pre-trial prisons ( *sledstvennye isolyatori*). Both donor and recipient countries are shy about revealing precise numbers and trends. ^15^ In response to a freedom of information request from one journal to Central Asian embassies about the number of their nationals incarcerated in Russia, all but one either declined to comment or failed to reply. ^16^ The exception was Tajikistan. Evidently, it is not in the interests of Central Asian states to publicise the offending behaviours of its migrant workers. In the Russian Federation, meanwhile, it has been business as usual where the publication of prison statistics is concerned. FSIN has never published data relating to prisoners' geographical distribution, nationality, ethnicity, race, and faith. 

The often-quoted figure that one-quarter of the 400–500,000 prisoners incarcerated in Russia today belong to one of 50 ethnic minorities must be approached with circumspection, therefore. We have no way of knowing from which of the official 158 ethnic minorities in Russia these originate and whether the figure includes foreign nationals from the near abroad who are officially resident in Russia. From reports based on official press releases, we know that between 2015 and 2020, the number of foreign nationals originating both from the near and far abroad who were incarcerated in Russia declined from c. 29,000 to 27,000 (see Table 1). This fall took place against a backdrop of a decline of 200,000 in the overall prison population, which gives a percentage point increase in the share of all foreigners in Russian prisons from 4.45% to 5.4%. ^17^

**Table 1.** Foreign prisoners in Russia’s penal institutions 2015–2020 (data provided by Russian media) ^18^.

2015	2016	2017	2018	2019	2020
28,714	29,822	29,397	27,780	26,000	27,000

FSIN has confirmed that the vast majority of foreigners incarcerated in the Russian Federation are citizens of the former republics of the USSR and over half of these are from Central Asia., Predominantly they are from Tajikistan and Uzbekistan, with the remainder from Ukraine, Belarus, Azerbaijan, Kyrgyzstan, Kazakhstan, Moldova, Georgia, Turkmenistan, and the Baltic states. ^19^ The largest group of foreign prisoners (8,002 of the total) in 2020 originated from Tajikistan, followed by those from Uzbekistan (6,362). In 2020, prisoners from Ukraine (but not including Crimea) came next on the list of foreign prisoners, followed by Azerbaijan and Kyrgyzstan. ^20^ The majority of foreign prisoners in Russian penal facilities are Muslims, although the proportions will undoubtedly have shifted since 2022 because of the Ukrainian War. In terms of their recorded offenses, more than 90% of foreign citizens in Russian penal institutions are serving sentences for serious crimes, including drug trafficking (39%), murder (12%), robbery (11%), intentional grievous bodily harm (8%) and rape (6%). ^21^

Compared with other jurisdictions, the share of foreign prisoners at approximately 5% of the total prisoner population, in the Russian Federation is modest. Inthe UK, for example, foreign nationals make up 11–13% of the prison population, and in the USA, the figure is 17.5% ^22^). In quantitative terms, therefore, the ‘foreign prisoner’ in the Russian Federation is a lesser ‘problem’ than in other jurisdictions. Nevertheless, the statistics on foreign migrants is an issue of serious concern to the authorities, as has been reflected in recent years by polices aimed at curbing the volume of arrivals from Central Asian. Meanwhile, the offending labour migrant has appeared as a figure to be feared or derided in FSIN’s journals, official print media, the blogosphere, YouTube, and social media. Muslim migrants who fall foul of the law, regardless of article in the criminal code that lands them in prison are believed to constitute a pool of potential recruits to violent jihad. ^23^ In official discourse and the public mind, therefore, the transnational Muslim prisoner is combined with offenders from the indigenous Muslim population - natives of the North Caucasus, Volga-Urals region and Russian-occupied Crimea – fuelling the impression of a sizeable Muslim presence in the overall prison population. Moral panic about prison jihad ( *tyuremnyi dzhikhad*) draws upon the discourse under Putin that understands traditional religions as the core of people’s identities in Russia and plays into xenophobic attitudes towards Muslim believers in general, regardless of their ethnic origin and school of Islamic law they might follow. ^24^ Charismatic leaders who preach violent forms of imported, ‘non-traditional’, Islam to the literally captive audience in Russia’s prisons. ^25^ The figure of the Muslim jihadist-in-the-making has displaced the principal Soviet-era Georgian ‘Thief,’ or *Vor*, as the dominant ethnic prisoner stereotype, although in the light of Russia’s invasion of Ukraine, this may well have already been displaced by the figure of the ‘Ukrainian fascist.’

In fact, of course, there are no reliable data available in the public domain of the total number of Muslims - domestic, foreign, and recently converted - in Russia’s prisons, let alone how many, among these, are followers of the different schools of Islam. Nor can comparisons be made of how numbers have changed compared with either the Gulag or the late Soviet period. For historical comparisons to be made, we would need a breakdown of the geographical origin of the Muslim prison population confined in the Russian Soviet Federative Socialist Republic (RSFSR) before 1991 who were first-generation migrants from the non-Russian republics, which we do not have. The lacunae in our knowledge of the Soviet-era practices concerning ethnic minority prisoners are numerous, including, for example, how many convicted prisoners in the RSFSR were returned to their ‘home republics’ to serve sentences and vice-versa (we know from interviews in the Estonia Soviet Socialist Republic (SSR) that serious offenders were generally transported to serve sentences, or for execution, to the RSFSR), and the ethnic content of the nationality statistics we do have. From the first post-Soviet calculations made using documents in the Central State Archive of the Russian Federation (GARF) the number of prisoners in the Gulag by national origin who were most probably Muslim was not disproportionately large compared with their background population. In 1937–1940, for example, Tatars, Uzbeks, and Kazakhs were underrepresented in the Gulag, and only Turkmen were over-represented compared to the recorded general population in the 1939 census. ^26^ For the later period, 1940–1951, Victor Zemskov’s calculations, again using the central archives, indicate a fall in the total number of Muslim nationalities in the Gulag camps and correctional labour colonies (ITLs) compared with Slavic and Baltic nationalities. ^27^

The Muslim prison population is not evenly spread across Russia’s vast penal estate. From research conducted for the GULAGECHOES project across Russia’s regions, we know that the share of foreign and Muslim prisoners varies widely between institutions and regions. ^28^ There are concentrations of Muslim prisoners in penal facilities in the North Caucasus, and Muslim Volga republics and in oblasts with Muslim enclaves across the country, which is to be expected. The greatest concentrations of transnational prisoners from Central Asia, however, are in pre-trial, remand prisons located in the main hubs of migrant labour, such as the metropolitan centres and regional capitals. For the past decade, the media has regularly reported on overcrowding in Moscow’s pre-trial detention facilities, with the principal culprit identified as the migrant labourers from the near abroad. Campaigns aimed at persuading the courts to substitute alternative preventative measures to remand in custody have been thwarted by the sustained high numbers of Central Asian migrants, who are considered a flight risk and for whom bail, or house arrest are inappropriate. Convicted offenders typically are transported to correctional facilities in the standard federal district catchment areas of the metropolitan remand prisons, although serious offenders and political prisoners can be sent large distances to colonies in the peripheries. In correctional colonies for convicted offenders in the oblasts of Moscow’s ‘penal catchment area’, according to our surveys with former prisoners, the percentage of Muslims can reach as much as 40% of the total number of prisoners in some institutions. Other places where transnational and Muslim prisoners are heavily represented are in the south of European Russia, the Muslim republics of the North Caucasus and Volga-Urals region, and in oblasts bordering the frontier with Kazakhstan in the West Siberia. These are institutions in which there is alleged to be an ever-present danger of prison-producing Islamic terrorists. ^29^

## Moral panic around prison jihad

Researchers in FSIN and Interior Ministry institutes trace the beginning of the process of transforming prisons into sites of Islamic radicalization very precisely to 1996, the year when Russia joined the Council of Europe. ^30^ This was when, so the critique goes, prisons were forced to open their gates to missionaries from European countries to demonstrate that they were meeting prisoners’ spiritual needs in line with Article 14 of the European Convention on Human Rights. ^31^ According to a three-stage model developed by FSIN researchers, the initial period of charismatic leaders’ penetration of prison in the late 1990s was followed by the formation of more than 300 radical prison jamaats which, from 2016, began to fuse into a single structure, with an estimated following of 10,000. ^32^ There is no way of checking these figures or revisiting the research on which they are based. Nor, indeed, do we know the number of terrorist acts committed in the past three decades by former prisoners, whether from right-wing nationalist groups, followers of Islam or others. 

The moral panic surrounding prison jihad conflicts with the official prison service discourse portraying its facilities as sites of ethno-religious harmony (the 21st -century version of the ‘friendship of nations’ trope). It insists that the long tradition of ethnic neutrality in inter-prisoner and prisoner-administration relations remains intact and, in fact, constitutes a bulwark against the foreign-imported Islam in its prisons. It is principally the Tajik and Uzbek migrants, not Russia’s indigenous Muslims, who are understood to be vulnerable to the teaching of charismatic recruiters to the cause of violent Islam. This is attributed to their failure to integrate into mainstream prison society because of their poor command of Russian and cultural-outsider status. Isolated from other prisoners, compatriot ‘families’ ( *semeiki*) and larger groups of believers consisting of ‘petty criminals and drug dealers’ from Central Asia, who come together to pray, are believed to be easy prey for charismatic leaders of radical schools of Islam. It was not simply a public relations gaffe, therefore, that when publicizing the ‘new’ punishment of “forced labour as an alternative to the deprivation of freedom” ^33^ *(prinuditel'nyy trud primenyayutsya kak al'ternativa lisheniyu svobody)*, FSIN stressed that this innovation in the criminal correction code is a means for Russia to reduce its need for imported, migrant labour on projects of nation-wide importance. The completion of the Baikal–Amur Mainline (BAM) railroad since the re-introduction of forced labour as a separate punishment modality can be achieved by deploying home-grown convicted offenders, obviating the need to import labour from Central Asia. ^33^ The principal means of combatting the threat of prison-jihad, it transpires, is to reduce the number of Central Asian in-migrants to Russia.

 Prisoner radicalization, especially along ethno-religious lines, is a difficult topic to research in any jurisdiction. Western research tends to rely on single-case examples of perpetrators of terrorist acts as a basis for modelling alternative pathways into and away from radicalization and violence. ^34^ Opinion in western jurisdictions is, in fact, deeply divided on the underlying issue of whether prisons are, in fact, breeding grounds for future jihadists but, with few exceptions, has not engaged with ‘prison jihad’ in Russia. ^35^ Researchers in FSIN have exclusively taken up the western literature supporting the idea of prisoner radicalization as primarily an imported phenomenon and takes comfort from the fact that ‘prison jihad’ is not unique to Russia. ^36^ In this way, the prison service not only can retain the myth of ethnic-blindness in its prisons but also can absolve itself of the charge that the conditions of detention in its prisons might be the root cause of Muslim prisoners taking oppositional positions to penal authority and to the broader power structure it represents. Indeed, the prison service describes its prisons as on the front line of defence against forces threatening the very existence of Russia. ^37^

 In this article, we take the first steps towards rectifying the various lacunae about the in-migrant Muslim prisoner in research on the Russian prison system. We acknowledge that the salience of ethnic and religious identities has increased since 1991 in the daily lives of the population of the Central Asian states, with Islam becoming a vital identity marker. Against this backdrop and in the context of a culturally alien prison system, it is reasonable to suppose that these circumstances will lead to ethnic and religious group formation among Central Asian prisoners and/or alignments with Russia’s Muslims. Using fieldwork conducted in Uzbekistan among former prisoners, we explore how the experiences of transnational Muslim prisoners who have become a presence in Russian correctional colonies shape, and are shaped by, the social order in prisons. This research focuses on small and intermediate group formation among prisoners and the importance in this process of individuals’ internalization of ethnic and religious norms. We examine how the resultant groups position themselves in relation to the ‘traditional’ licit and illicit hierarchies in Russia’s prisons, and the factors that affect this positioning.

Our findings are that migratory processes post-1991 of large numbers of ethnic Muslims from Central Asia who are legally classified as foreigners and understand themselves as such, is a new phenomenon associated with the break-up of the USSR. The conviction and imprisonment for criminal offences of these foreign migrants, we argue, has transformed some Russian penal institutions into plural legal environments characterized by patterns of coexistence and conflict between the rules of formal and informal power nodes. We describe the changing geometry between the ‘regime’ of colony administration, the so-called ‘thieves law’ ( *ponyatie*) associated with the traditional prison sub-culture, and Sharia law associated with groupings of Muslim prisoners. Our conclusion is that ethno-religious solidarity norms can flow through each of these power nodes, confirming the salience of religious and ethnic differences in inter-prisoner and prisoner-administration relations during the post-Soviet era. However, we find no evidence that these processes necessarily lead to the formation of trans-penitentiary radical jamaats dedicated to violent jihad.

### Secondary sources on foreign prisoners in Russia

There is extensive literature on migration in Russia covering various topics which reflects Russia’s emergence post 1991 as a key global migration hotspot. ^38^ However, even though there are more than 11 million foreign-born individuals temporarily resident in the country, a proportion of whom commit criminal offences, there has been little academic research published on the pattern of foreigners’ and ethnic minority incarceration in Russia since 1991. This is true even of the academies subordinated to the Federal Prison Service that have their own research departments and of other research institutions subordinated to central power ministries. ^39^ A keyword search undertaken in 2020 e-library.ru, the largest Russian information and analytical portal in science, technology, and medicine, showed a significantly large increase in articles published with the keywords ‘religion+extremism’ from 1990-2000 to 2015-2020 (from six to 2,374 articles using these keywords) and with the keywords ‘religion+prison’ (from 414 to 8,481). ^40^ While the expansion of the literature indicates that ethno-religious issues in the penal context have become a topic of interest among experts, follow up reading of a selection of the articles shows this literature to be repetitive, reliant on information from central and regional media sources, and derivative of a narrow range of western scholarship on prison radicalization. Following western literature, experts in FSIN’s academies assume a linear progression of Muslim prisoners’ religious rituals and group-based ( *jamaat*) praying practices to religious extremism. The literature is largely silent on how imprisonment impacts upon the self-identification and experiences of Muslim prisoners from Central Asian republics and of the latter’s impact, in turn, on the traditional social order and power hierarchies in Russian prisons.

The paucity of research on Muslim prisoners can be explained by the sensitivity of debate about nationalism in Russia, especially concerning the criminal justice system's treatment of migrants and ethnic minorities. ^41^ It is challenging to collect empirical data on transnational prisoners' carceral conditions and experiences and to assess their vulnerabilities compared with majority Russian nationals. Human rights activists often face restrictions on gaining access to prisons and non-governmental organizations (NGOs) have been reluctant to prioritize publicizing information about discrimination against foreign Muslim prisoners, unless they are high-profile prisoners of conscience. ^42^ Occasionally, print and internet media report on fabricated criminal charges and incidents of torture and inhuman and degrading treatment of Central Asian prisoners in Russia. ^43^ Meanwhile, there has been a complete blackout of information from the side of FSIN One exception to the failure of academe to address the question of ethnic and ethno-religious relations in Russian post-Soviet prisons is an article published by Ivan Peshkov in 2015 in which he argues that penal space in Russia continues to remain ethnically- and religiously-neutral despite the ever-growing anti-migrant sentiments and manifestations of inter-ethnic tensions in the wider Russian society. ^44^ Rather than pointing to the internalization by prisoners and administrations of deeply embedded ‘friendship of nations’ approach to ethnic and, now, religious differences as the explanation, Peshkov instead attributes the apparent lack of ethnic conflict in Russian prisons to a legacy of the dominance of Soviet criminal subcultures and. long-term effects of the Gulag system that subjected all prisoners to the norms of criminal culture regardless of their ethnic belonging. The Soviet criminal subculture, combining the idea of ‘the friendship of peoples’ with an ideologically non-ethnic prison hierarchy system, limited the overt expression of ethnic solidarity and established a criminal cosmopolitanism model which became a standard operating procedure for all inmates. The heirs to the *vory-v-zakony,* Peshkov argues, follow their forebears in suppressing the emergence of ethnic solidarity in correctional colonies for fear of them coalescing into alternative centres of power. He argues that the Soviet policy of ‘cosmopolitan universalism’ had become a deeply embedded operating principle for criminal sub-cultures in their management of ethnic diversity in correctional colonies and has ensured their enduring superiority over ethnic and religious solidarity. For Peshkov, reproduction of Soviet model of “cosmopolitan universalism” not only gives prison communities a possibility to manage ethnic diversity but also ensures the superiority of the criminal subculture over ethnic and religious solidarity. In a nutshell, Peshkov concludes that “ethnic situation in prisons resembles the USSR rather than today’s Russian Federation” and “regular Soviet prison is still there.” ^45^

 The academic literature on the thieves-in-law in both Soviet and post-Soviet prisons is divided on the presence and power of criminal sub-cultures at different times. In the 1990s, for example, when there was more Soviet penal scholarship than now, it was argued that the traditional thieves sub-culture had been displaced by prison gangs organized around the drugs trade. Others observed that fundamental changes were taking place in the *modus operandi* of the Thieves-in-Law, as they migrated out of the prisons. ^46^ Regardless of different schools of thought about whether the power of traditional thieves’ understanding or *ponyatie* in prisons has survived the existential challenges of transformations in prisons since the collapse of the Union of Soviet Socialist Republics (USSR), criminal subcultures still represent themselves as heirs to the traditional Thieves-in-Law. The dominant questions today revolve around issues of thieves’ authenticity and performance, as much as their power and dominance of prison society. ^47^

### Learning the glossary of the Russian penal space

Human rights advocates of penal reform use the term ‘neo-gulag’ to describe the current prison system in the Russian Federation. Underpinning this label is the proposition that today’s penal institutions in Russia have carried forward legacies from Soviet-era correctional labour colonies, which were gestated in the Stalinist Gulag. Soviet legacies in today’s prison system have been discussed and critiqued in the small body of English-language literature on the prison systems of all the Soviet and East European communist successor states. ^48^ For the purpose of our analysis, the main carry-over feature of the Soviet-era penal institutions influencing the choices made by transnational prisoners from Central Asia is the architecture of the correctional colonies in which the majority of transnational prisoners serve their sentences, which accommodates prisoners communally. ^49^ The communal living block, together with the tradition of prisoner self-government also inherited from the Soviet era, create physical and social space for interactions between prisoners that remain largely outside the day-to-day purview of prison personnel. ^50^ In most jurisdictions, the order in penal institutions is maintained by negotiation between prison officers and prisoners, individually or collectively, albeit with ultimate power lying with the institution because of its right to use physical force. ^51^ The power geometry in prisons varies across jurisdictions, however. The expansive physical and transactional space that Russian correctional colonies inherited from the USSR marks it out from other jurisdictions in the developed world and has long provided the context for the emergence of powerful prisoner sub-cultures. These existed in the Soviet era but entered a new lease of life in the years of crisis or ‘ *bespredel’* in prisons in the 1990s. The correctional colonies in which transnational prisoners are confined are referred to as the ‘ *zona*’. ^52^ In terms of power hierarchies and governance form, penal zones are popularly described as falling into one of three main types: ‘black’ ( *chernaya zona*), ‘red’ ( *krasnaya zona*), and ‘regime’ ( *rezhimnaya zona*). ^53^ Each colour code implies a particular set of power relations in respect of which transnational prisoners have to position themselves. In our discussion of the ‘lexicon’ of the power geometry of colonies below, we are using the meanings the research participants’ vest in the vocabulary used in the particular institution(s) in which they were confined. These do not necessarily describe how the decisions affecting prisoners are, in reality, made in prisons and correctional institutions, which is better understood by reference to more complex governance models. ^54^ There is, in fact, a significant variation in the governance of penal institutions in Russia between and within regions When prisoners talk about power, they are not referring to it in its official or legal sense; they fully understand that ultimate power over their lives lies with the penal authorities, as the agents of the Russian state. The power relevant to prisoners is social and relational. The colour-coding of colonies refers to the relationship as understood by prisoners between the hierarchies or social groupings that determine and enforce the rules for the conduct of everyday life within the prison environment.

‘Black zones’ are those in which the balance of power lies is believed to lie with the leaders and illicit power hierarchies of the criminal world prison sub-culture. Most prisoners label these as the *Vory-v-zakone* or Thieves-in-law and subscribe to the informal thieves law or ‘understanding’ ( *ponyatie*). ^55^ With some exceptions, the Russian version of prison argot is used by transnational prisoners, regardless of their knowledge of the Russian language. The hierarchy of ‘thieves’ in a black colony is headed by the *polozhenets* or *smotriashii* (understood as the local representative of a crowned *Vor*) and *barashniki* (the principal authority of individual barracks and dormitories where prisoners live). *Polozhentsi* and *barashniki* play decisive roles in a zone’s everyday governance and ‘the rules of the game’ with which prisoners must comply. In black colonies, formal prison management structures have a limited role regulating prisoners’ everyday lives and routines. Cigarettes and tea serve as currencies in prisoners’ daily transactions and relations. Prisoners in black colonies can indulge in card games ( *qimor*) at night and are expected to contribute to a mutual assistance fund ( *obshak*) administered by the sub-culture, and they are expected to take part in protests involving self-harm or hunger strikes, as directed by the sub-culture’s authority figures.

Prison management and power relations are different in ‘red zones’, where, according to prisoners’ narratives, formal prison management structures consist of the prison governor ( *nachal’nik kolonii*) and deputy governors, each heading up one of the facility's functional departments and other personnel. Prison staff are referred to colloquially as the *menty* in Russian; a term also applied to the police and armed militias. In red zones, the administration usually relies on the assistance of ‘activist prisoners’ ( *aktivisti*) to help maintain formal order. The activists are either forcibly recruited by the internal security operational officers ( *operativniki*) or who volunteer their services to run prisoner self-organization committees, which are the officially legitimized groupings responsible for the day-to-day running of a detachment dormitory. In everyday speech, activists can also be called ‘bitches’ ( *suki*) or ‘reds’ ( *krasnie*). A sub-group of these are informants recruited to secretly report on fellow prisoners to the operational officers. Activists are hierarchically ordered, with every dormitory having a prisoner-in-charge ( *zavkhoz, dneval’nyi*) and their deputies down to rank-and-file members of self-organizing committees. This official prisoner hierarchy is supposed to be a counterweight to the sub-cultural hierarchy. Effectively, they exist to assist the prison administration enforce prisoners’ compliance with internal colony rules as the primary code of conduct in their daily life and routines. Given the weak influence of the criminal world, prisoners serving sentences in red zones are compelled to participate in interventions ( *meropriyatii*), comply with the official daily timetable, and work, as ordered, in the prison’s industrial and agricultural zones. 

In addition to black and red zones, there are also colonies prisoners refer to as ‘regime zones’ where power is more evenly shared between the criminal sub-culture and the formal prison administration. In a regime zone, both sides make concessions to each other and jointly determine the codes of conduct for prisoners. As a result, regime zones have a hybrid form of governance. In a regime zone, for example, prisoners might agree to wear the prison uniform ( *roba*) during the day, as is obligatory in red zones, but engage in night-time card games, consume narcotics, or use smartphones and the internet which are prohibited by regime rules, but to which the personnel turn a blind eye. In regime zones, prisoners can decide for themselves whether to work, the decision about which normally depends on their financial situation. Among the prisoners interviewed, a *polozhenets* in a regime-colony was normally dubbed an ‘orange thief’ ( *apelsinovii vor*) because of their willingness to negotiate with the authorities and their inability to enforce fully the basic tenets of the thieves law. ^56^

All prisoners arriving in a correctional colony are confronted with choices that will affect their daily routines, status in prisoner society, and dormitory or detachment ( *otryad*) placement. The often-long periods spent in pre-trial detention is a critical period of education for first-time offenders about the ‘folkways’ of the penal world. By the time they arrive at their destination correctional colony, most prisoners already have decided, or have had the decision made for them, what place they will occupy in relation the existing power hierarchies. The latitude and the factors they must consider in making their choices depend upon the ‘colour’ of the zone in which they will serve their sentence. In some zones, there is a clear functional division of labour between barracks and detachments in which new arrivals will be placed. These can consist of barracks for working prisoners (the *rabochii barak*), those for prisoners who decline to work or are unfit or too old to do so (the *nerabochii barak*), those for prisoners with an interest in the thieves law ( *zainteresovannie and poryadochnie muzhiki, i.e., lyudskoi barak*), those for the activists (the *krasnii barak*) or some hybrid of these. ^57^ While most prisoners can have some influence over their placement, there is a range of behaviours and types of offense that remove choice. This applies to prisoners convicted of sexual offenses, including offenses against children, and/or individuals who are believed to engage in non-traditional sexual practices. These prisoners make up the lowest caste in prison society and are usually confined to separate spaces in colonies, either in barracks reserved for the outcasts ( *opushennie/obizhennie*) or in specially designated bunks in barracks in a hybrid detachment or barrack.

As Russian penal space has had to accommodate the arrival of transnational prisoners, new power hierarchies and social groups based on ethnic and religious lines have also emerged. Popularly, it is supposed that there are ‘green’ colonies and ‘green’ detachments or barracks to set alongside the ‘black’ and ‘red,’ and ‘regime’, in which Muslim prisoners dominate. In fact, the current policy in FSIN is to disperse Muslims among other prisoners unless they are convicted of terrorist offenses. In the latter case, the prisoner is confined in one of a small number of special regimes colonies or prisons that have cellular accommodation. Nevertheless, as we have already observed, the number of Muslim prisoners in some regions is such that they can form intermediate and higher-level social groupings or jamaats based on the tenets of Islam giving them a significant local presence in specific colonies. These groupings are led by an imam (religious leader) chosen by the prisoners because of their knowledge of Islam and Sharia law. These intermediate social groups are neither part of the traditional criminal subculture nor part of the administration-sanctioned hierarchy of activists. Instead, they position themselves as an autonomous community within prison society, membership of which is restricted to Muslims who commit to living and organizing their daily life according to Sharia Law. They invariably are ‘constructed’ as sub-cultural groupings by colony administrations, although they are not necessarily oppositionist, as a matter of principle.

In some cases, these Muslim groupings have an ethnic content in that they are composed exclusively of non-Russian Muslims from Uzbekistan, Tajikistan, and Azerbaijan, sometimes together with ‘non-traditional’ Muslims from Chechnya and Dagestan. As we will show in the next section, these new ethno-religious formations impose their own norms of conduct on Muslim prisoners and compete for influence vis-à-vis the traditional criminal subcultures and prison administration. In doing so, insert a new dynamic into prisoner society. The process of group formation. Group formation among the transnational prisoners from Uzbekistan appears to be a function of their number and their foreigner status, which excludes them from the family of diverse Russian nationals. As we show below, the processes of exclusion and inclusion can shape the transnational prisoners’ choices as they position themselves in relation to the changing power geometry in Russia’s prisons today

## Methods

A combination of foreign agents’ legislation, FSIN’s suspicion of independent scholarship, and COVID-19 restrictions from 2020 (many of the latter remaining in place at the present time) have restricted researchers' access to serving prisoners. ^58^ This has affected the GULAGECHOES project, directing us to pursue our study of the migrants’ experiences of incarceration in Russia to former prisoners who were repatriated to Uzbekistan after the conclusion of their sentence. The data for this article, accordingly, are drawn from two periods of field research in the Ferghana Valley of Uzbekistan in January and September 2020 and revisiting interview data from prior extended ethnographic fieldwork in Moscow between 2014 and 2018. The fieldwork took place according to the ethical protocols approved by both the European Research Council and University of Helsinki Ethics Committees, and were monitored by the GULAGECHOES Ethics Advisory Board constituted in 2019. ^59^ In Uzbekistan, the project was affiliated with the Academy of General Prosecutor’s Office, which provided a local ethics clearance in accordance with the Memorandum of Understanding signed between University of Helsinki’s Aleksanteri Institute and the Academy of General Prosecutor’s Office of Uzbekistan in 2019.

Data collection drew on the first author’s unique ‘ethnographic toolkit,’ consisting of his command of the Uzbek and Russian languages, a strong network of contacts developed over a number of years in the migrant community in Moscow, and his cultural-insider status. ^60^ Existing contacts allowed him to gain access to informal networks of Uzbek nationals. The selection criteria included men and women with varied criminogenic profiles and lengths of sentence, who had served one or more sentences in Russian penitentiary institutions between 1991 and 2020. 29 research participants were recruited with whom in-depth, ethnographic interviews were conducted. In identifying and recruiting research participants, the first author relied on the assistance of a gatekeeper, an ex-prisoner who served a prison sentence in Russia and was well-connected with Uzbek ex-prisoners who had served one or more sentences in Russian penitentiary institutions between 1991 and 2020. Our gatekeeper, possessing knowledge of the unwritten rules and moral codes among the ex-prisoner community, provided initial information to our interviewees about our research project based on our information sheet. This meant that our interviewees had sufficient time to consider whether or not to participate in our study and to prepare any questions troubling them. The gatekeeper contacted 38 potential research participants of whom 29 agreed to participate in the study. During the first period of fieldwork (January 26 -February 8, 2020), 10 interviews were conducted. These helped the first author explore the topic as it was a new research field to him. By revealing the novelty of the research field to his interlocuters, he was able to neutralise what otherwise would have been an unequal power balance between himself and the research participants, who were able to ‘school’ him in the details of serving sentences the Russian prisons. ^61^ This helped to build rapport. ^62^ The interviews allowed him to obtain an initial understanding of the Russian penitentiary system, as well as to build his vocabulary of the prison argot used by prisoners. During the second period of fieldwork (August 28-September 23, 2020), 19 interviews with former prisoners were conducted. While the first period served as an introduction to the subject and field, the second period enabled issues to be followed up that had surfaced in the first exploratory field trip and gain an in-depth insight into the issues of ethnicity and religion in Russian penal institutions through the experiences and narratives of Uzbek ex-prisoners.

Our methodological choices and data collection strategies were largely driven by our ambition to trace the biographical trajectory of each participant in the research. This was achieved by dividing the interview questions into four stages consisting of: (i) the period before the arrest, (ii) the period of arrest and detention, (iii) all stages of serving a sentence, and (iv) the period after release. The aim was to analyse power geometries, emotions, and affective repertoires associated with the individual prisoners’ religious and ethnic identities and how these power dynamics shape their carceral experiences and trajectories, given the stage in their family and personal life-cycle. The open-ended interview format, rather than the closed interview, was best suited to this aim because it places emphasis on power shifts and emotional labour in the interview context, allowing the researcher to understand the nuances of the data, and providing relevant personal information about the research participant, as well as facilitating in-depth insights into the interview process, the subjects, and the nature of the topics discussed. ^63^

Based on these considerations, prompt questions were designed to elicit narratives and self-reflections from the interviewees, encouraging them to recount the experiences and events that shaped their penal journey, allowing them to pause, think and reflect on their experiences. The interviews covered a range of topics that surfaced in the research participants’ talk in response to the invitation to talk about their journey from their life prior to arrest through the various stages of detention to release, and their life since. The narratives of their penal journey allowed us to examine how race, age, gender, citizenship, ethnicity, and religion affected how transnational prisoners from the ‘near abroad’ positioned themselves in relation to the informal and formal power structures they encountered in prison, and how this was influenced by the identities constructed for them by the prison administration and other members of prisoner society. Early in the fieldwork, it became apparent that ethnicity and religion ran through the informal rules and norms of behaviour that regulated prisoners’ everyday lives. This focused our attention on the process of ethno-religious ‘awakening’ that research participants experienced during their carceral journey. The interviews generated a rich stock of empirical material on which we could draw to interrogate the stereotypes or experiences of the transnational prisoners in Russian prisons.

All 29 interviews were conducted face-to-face through a conversational process, which lasted from 45 minutes to three hours, depending on the interviewees’ available time, location, and preferences. At the outset of each interview, each research participant was informed that the content of interviews would exclusively be used for academic purposes. The first author started each interview by reiterating the details of the research, asking each participant if they had questions about the aims and objectives of the research, the researcher’s background, confidentiality, safety, and data protection issues, and the data collection process. The participants were told that they could terminate the interview at any stage and could decline to answer individual questions. All interviews were numbered and audio-recorded after obtaining the interviewees’ consent. The research participants' preferences determined the choice of places for the interviews, with the result that the interviews were conducted at various different types of location in the Fergana Valley. These included private rooms of cafés and restaurants, choykhona (teahouses), guzar (neighbourhood community meeting spaces), private houses and apartments, and bazaars. In addition to audio recordings, interviews and observations were documented in field diaries. The most obvious identity markers from audio-recorded interviews were removed whilst still in the field. Audio-recorded interviews were transcribed within one month after the completion of the fieldwork. Considering our interviewees’ requests, we decided to use pseudonyms (instead of numerical codes) when presenting interviewees’ penal life-stories.

The use of an empathetic interviewing style was crucial in building trust and rapport with interviewees. At the beginning of the interview, it was made clear to each participant that the aim of the interview was not to delve into their “delyuga” (why they/ex-prisoners committed a crime/ended up in prison), but rather to examine power hierarchies, ethnicity, and religion in Russian prisons through the lens of their penal experiences and life trajectories. They were also reassured of the project’s value-and-judgment-free stance with regard to situations and motivations that resulted in their imprisonment. When preparing the interview guidelines, a primary concern had been to formulate and structure questions in a friendly way. Therefore, the interview began with questions focusing on the research participant’s life and experiences in Russia as a labour migrant before the arrest. The final part of the interview returned to questions about research participant’s life ‘in freedom’, after release focusing on their experience of the re-entry and reintegration processes into Uzbek society. Hence, our interview started and ended with questions that were not directly related to carceral experiences. Finally, the willingness of the interviewer to use, and be corrected in his use, of prison argot during the interview helped build rapport. The success of these various strategies for building trust bore fruit, to the extent that the majority of research participants talked openly about their carceral experiences without, apparently, feeling the need to recourse to lengthy justifications of the actions and choices that had led up to their imprisonment, avoiding or refusing to answer what at times were difficult questions.

Using the narrative of the penal journey of one Uzbek former prisoner, whom we have decided to call Farhod, we can show how the processes of exclusion and inclusion can shape the transnational prisoners’ choices as they position themselves in relation to the changing power geometry in Russia’s prisons today. We are not suggesting that Farhod’s story can be generalised across the prison estate, but rather we use his example to help identify the factors that shape the choices in-migrants from Central Asia must make about their allegiance to the existing hierarchies they find on arrival at their destination colony. Farhod was interviewed in 2020, shortly after he was returned to Uzbekistan having served a five-year prison sentence in Russia

## Results

### Navigating the plural power hierarchies in Russian Prisons

Farhod is a male, 37-year-old ethnic Uzbek from the Fergana Valley. He served his sentence in a strict regime ( *strogii rezhgim*) correctional colony for first time offenders between 2015 and 2020. Farhod had migrated to Russia in 2010 to join his father, who owned an Uzbek eatery in the wholesale bazaar in a regional centre in an oblast 625 kilometres southeast of Moscow, before labour migration became a mass phenomenon for Russia and Uzbekistan. This restaurant was a hub for Uzbek entrepreneurs ( *rossiychilar*) who traded Uzbek fruits and vegetables with Russia. Thanks to his father’s networks, Farhod rapidly integrated into the local labour market and everyday life in Russia. Under his father’s protection, his life was pleasant. He earned around 50 USD a day helping his father, an income more than sufficient for him to enjoy life in the city. However, Farhod’s fortunes changed as he was drawn into the world of drugs. In the early spring of 2015, he was stopped and frisked by an officer of the Special Designation Militia Detachment (OMON: *otryad militsii osobogo naznacheniya*) and was found to be carrying hashish and heroin. He was held in pre-trial detention for eight months before coming to trial. He received a five-year sentence and was transported to a strict regime colony some 500 kilometres away.

According to Farhod’s estimate, there were 3,000 prisoners in his correctional colony. Ethnic Russians were the dominant group, but there were sizeable numbers of Chechens, Dagestanis, Ingushes, and Tatars. He calculated that approximately 15-20% of prisoners were foreigners, primarily Tajik, Uzbek and Kyrgyz nationals from Central Asia, with smaller numbers from Azerbaijan, Armenia, Georgia and Abkhazia. There was also a handful of prisoners from Ukraine and Moldova, plus four men from the Middle East, imprisoned for drug-related offenses. Prisoners in the zone were assigned to a detachment ( *otryad*) a collective group of up to 100 prisoners occupying part of one floor of a barrack-type building. The members of Farhod’s detachment had their own communal dormitory, a common room, a kitchen/pantry, bathroom, and cloakroom, and was in one of several barracks in the colony. ^64^ According to Farhod the barrack in which his detachment was located held 400 prisoners of whom ‘at least’ 70 were Muslims. 

Farhod’s imprisonment coincided with attempts by the prison service to address high recidivism rates in the Russian Federation by separating repeat from first-time offenders. The thinking behind this reform launched in 2010 was that internal regime rules would be easier to enforce with populations of prisoners who had not been indoctrinated into the traditional prison sub-cultures. Separating neophytes from recidivists would allow the former to benefit from interventions aimed at encouraging them to desist from crime after release. ^65^ However, it was obvious that contrary to the aims of the policy of separation, the colony in which Farhod served his sentence was firmly ‘black’ with prisoner governance lying with the prison sub-culture, under the leadership of a *polozhenets*. Rather than his everyday life being regulated by the formal rules of a strict regime colony, which would have subjected him to an inflexible daily timetable, he was able to enjoy a high degree of freedom of movement and association inside the prison’s perimeter fences. He also had access to means of uncensored communication with the outside world, so long as he had the resources to benefit from the shadow market run by the criminal sub-culture. Like generations of prisoners before him, Farhod had to learn the informal folkways of the colony quickly and position himself in relation to other Muslims, the criminal sub-culture, and prisoners in the colony who might be working for the administration. 

### Islam and prisoner hierarchies

Muslims constituted a minority group in Farhod’s correctional colony, but they were better organized and united than the majority Russian population. Furthermore, thanks to the fact that the criminal sub-culture, which was multinational in composition, was headed by a Chechen *polozhenets*, Muslims had more influence and voice than non-Muslims with the result according to Farhod, that his was a “Muslim-dominated zona”. The Chechen wore two hats. On the one hand, in his position as a *polozhenet*s he was a local representative of the thieves-in-law and made sure that the ‘understanding' ( *ponyatie*) would be reproduced and enforced in the zone’s everyday life. On the other hand, he was a Muslim which meant that he also had a religious duty to protect the interests of Muslims in the zone, making sure that they received decent treatment. While heading up the everyday activities of the criminal sub-culture in the internal market in drugs and cigarettes, nightly card games, and collection of the *obshak*, the Chechen *polozhenets* also ensured that the varied needs of the Muslim inmates were met. These included arranging for Muslims to have access to halal food and for any who wished to pray five times a day to be able to do so. ^66^ Thanks to the *polozhenets*’ efforts, the prison administration allowed Muslim prisoners to use one of the unused barracks in the colony as a mosque for collective prayers. The imam, chosen from among the prisoners, was an Uzbek from Tajikistan who was known as ‘Sheykh’ among prisoners due to his rich knowledge of Islam and oratory skills.

The mosque in the colony was permanently open for prayers, except for the *bomdod* (morning prayer before the sunrise). Muslims usually read jamaat *namaz*; that is, they prayed together, in a gathering/in a large group setting. Friday prayers were the most attended religious event. At a minimum, Farhod said, 100 people attended Friday prayers. The primary role of Sheykh was to lead prayers, tell different *hadith* (records of the traditions or sayings of the Prophet Muhammad) before the prayers, and advise Muslims on how to follow the five basic tenets ( *farz amallari*) of Islam. ^67^

These daily prayers at the mosque transcended ethnic differences between the various domestic and transnational groups of Muslim prisoners, which created strong solidarity among them. Regardless of their ethnic group and geographic origin, Uzbeks, Tajiks, Chechens, Dagestanis, Azerbaijanis, and Tatars were united during prayers. The mosque was used not only as a gathering place for prayer, but also served as a site of support and networking among Muslim prisoners from different barracks. This was particularly helpful for young and newly arrived Muslim prisoners, giving them moral support, advice about zone rules, and mentoring in necessary survival skills.

In addition to daily prayers, Islam was also incorporated into the everyday life of Muslim prisoners. As it is not allowed to eat pork in Islam, many Muslims did not eat in the zone’s canteen because there was no guarantee that meals prepared in the zone were halal; it was rumoured that the same cooking pots were used for pork and beef. Instead, Muslims gathered money from the community and cooked separately in their barracks. During Ramadan, Muslims were able to fast together and share morning ( *saharlik*) and evening ( *iftar*) meals.

Engaging in daily prayers and Ramadan opened many doors to Muslims. As each prayer requires a separate cleaning and purifying ritual ( *tahorat*), special bathing and cleaning facilities were arranged in several bathrooms. But these facilities were only accessible to Muslims, and non-Muslim prisoners were not allowed to use them. In short, Muslims managed to organize daily life and religious practices to suit their needs in the colony, even though they were numerically in the minority. In theory, of course, the right for minorities to observe their cultural and religious rituals is guaranteed in the Russian Federation criminal correction code. ^68^ Still, it was obvious from the interviews conducted with transnational Muslims, practice across the penal estate is uneven, with human rights NGOs reporting many instances of failure to respect or to abuse, Muslim traditions. ^69^ Farhod believed that discrimination against Muslims in his colony was prevented because of the unity among Muslims and the strong solidarity they built based on Islamic principles. He contrasted this situation with the majority Russian population, who had no such unifying ideology, which, he said, was also true for other non-Muslim transnational prisoners.

Farhod noted that the influence of Muslims in the zone had increased during the time he served his sentence to such an extent that Russian prisoners had begun to use Islamic and Arabic modes of address when interacting with Muslims, such as “ *Assalamu Alaykum,”* “ *Inshallah,”* “ *Allahu Akbar.”* There were also Russian converts to Islam, which Farhod attributed to their desire to gain access to the material and psychological support that came with belonging to the supportive community. Furthermore, by showing respect to Muslims, non-Muslim prisoners were able to share the produce parcels Muslim prisoners received from their contacts outside the prison. A measure of the elevated position of Muslims in the zone was demonstrated at festivals such as Eid, when all prisoners, regardless of faith and ethnicity, were treated to the traditional Uzbek meal of *plov*. In all these instances, the role of the Chechen *polozhenets* was crucial, as he constantly lobbied and defended the interests of Muslims with the administration and quashed any objections that might arise from other prisoners inside this particular zone. 

### Surveillance of Islam by prison staff

As the influence of Muslims in the zone increased during Farhod’s incarceration, the prison administration gradually intensified its surveillance of religious practices. This surveillance focused mainly on Muslims from Dagestan, who ascribed to the Wahhabi and Salafist interpretations of Islam. The administration’s main concern was that these radical interpretations of Islam would attract other Muslim prisoners. and set them on the path to violent radicalisation. The administration began closely monitoring the religious activities of all Muslims in the colony. These measures included instructing Sheykh on what he should say during daily prayers, banning specific texts that were considered extremist, and using CCTV to monitor who entered the mosque. Of the approximately hundred people who attended Friday prayers, 40 read *namaz* five times a day. Therefore, according to Farhod, they could be considered more devout in their practice of Islam. The colony’s surveillance systems easily identified them by the regularity with which they were picked up on the CCTV entering the mosque. 

While modern surveillance techniques are employed in all Russian correctional colonies today, reliance is still placed on the recruitment by the operational officers of informants to give regular updates on, for example, the religious practices of co-prisoners. All Muslims knew that there was always one informer among them who reported to the prison administration on their conversations and on what was happening in the mosque. As a consequence, Muslims were careful about openly expressing their religious ideas. Meanwhile, the internal placement policy in the zone aimed to avoid over-concentrations of alleged radical Muslims by distributing them between detachments and barracks

The Chechen *polozhenet*s also played a role in suppressing the spread of radical ideas among Muslims in the colony. In an explanation that qualifies Peshkov’s thesis adumbrated above, Farhod attributed the *polozhenets*’ apparent support of the administration’s deradicalization strategy to his determination to protect the more moderate Muslim believers from the risk of the closure of the mosque, which he believed would have been a consequence of the more radicalized Dagestanis spreading their ideology. There was, in other words, a coincidence of interests between the prison administration and Chechen *polozhenet*s on this issue. The example of this type of mutually supportive negotiation between prisoner leaders and the administration speaks more generally to the role that ethnic identity can play in the power geometry in Russian penitentiaries and, more specifically, in the case of black colonies, of the crucial role of the ethnicity of the leader of the sub-culture can play in protecting the interests of specific ethno-religious groups in Russian prisons today. In this particular case, therefore, the existence of a critical mass of Muslims prisons in a colony can be understood as reinforcing the power of the traditional criminal sub-culture in colony governance. Whether this is repeated in other colonies depends crucially upon the ethnic and religious identity of sub-cultural leaders and the colour of the colony. Hence, different power relations between Muslims and the criminal subculture can be observed, depending upon the concentration of Muslims. We can take the example of one of the correctional colonies, let us call it correctional colony ‘X’, in a region 1000 km northeast of Moscow. According to one research participant who served a prison sentence there between 2016 and 2019, a group consisting of 35 devout Muslims (to which he belonged) avoided any involvement with the criminal subculture. As a rule, every month prisoners were expected to make monetary or non-monetary contributions to the common pot. If such contribution was intended for the expenses of barrack (e.g., tea, sugar), Muslims contributed with both money and labour. However, if the monetary contribution was intended for the *Vory* or for expenses such as cigarettes, Muslims refused to contribute. They stated that these practices are haram (sinful) contradicting the Koran and prophet Muhammad’s sunnah. This meant that Muslims contributed only if the money gathered was used for halal (permissible, pure) practices. Of course, the thieves’ *polozhenets* and *barashniki* did not like Muslims' attitude, but they had limited leverage given that Muslims were a close-knit group organised around Muslim communal values.

### Ethnic solidarity

Another aspect of the correctional colony on which Farhod commented was the support and solidarity networks upon which Muslim prisoners could draw by virtue of their ethno-place identity. These networks existed in the colony, despite the attempts by the administration to suppress group formation among prisoners based on ethnicity or region of origin. The expression ‘ *zemliachestvo-blyadchestvo’,* which roughly equates to saying, ‘supporting your countrymen is equal to prostitution’, was widely used by the administration and some prisoners to condemn the practice. However, in various ways, race, ethnicity, place, and geographic origin flowed through the interpersonal relations of prisoners on a day-to-day basis. Conversations, networking, and mutual support among prisoners were based on ethnic identities and found expression in the formation of families ( *semeiki*) - the small, lowest scale, horizontal social formations - that exist in most correctional institutions in Russia. Farhod identified the types of conversations that cemented group formation, such as consensus around historical events or prominent national heroes. Examples of historical events that conferred status upon ethno-religious groups, included the 1920s Uzbek Basmachi uprising against Russian Imperial and Soviet rule which represents Uzbeks as fearless opponents of the state power. References to Tamerlane, founder of the Timurid Empire in the 14 ^th^ century, and now considered the founding father of the contemporary Uzbek state, was also a common reference point for Uzbek prisoners, further enhancing their reputation in prison as a fearless and rebellious nation. This view was endorsed by the Chechen *polozhenets* and *barashniki*, occupying the highest positions in informal power hierarchies in Farhod’s zone. Tajik prisoners made a claim to a different heritage, emphasizing their intellectual contribution to human civilization by reference to the scholars of the Islamic Golden Age, such as Al-Farabi, Ibn Sina (Avicenna) and Al-Khwarizmi were Tajik/Persian who ‘invaded the world with the pen’, in contrast to the ‘barbaric Turkic groups’. ^70^

Having a strong cultural capital and glorious past, it turns out, was very important in determining social and ethnic group formation and played a role in the self-identification of many of the research participants. History and cultural capital were key markers of the identity, especially under opaque prison conditions where inmates have to constantly negotiate their status and standing in relation to existing power hierarchies. This was not unique to Muslims. Farhod recalled that Georgian prisoners also used history to enhance their status in the society of prisoners, referring both to the Georgian origin of Stalin and of the ‘authentic’ thieves-in-law. Farhod’s examples were drawn exclusively from the minority ethno-religious groups in his colony. As the empirical data we collected for this study mainly focuses on Uzbek prisoners’ experiences, we do not know the extent to which these types of ethnic self-ascription and boundary-marking were also common among the majority of Russian prisoners or are associated exclusively with minority prisoner groups. But they are an indication that ethnicity clearly plays a role in group formation in the post-Soviet prison.

Farhod’s narrative confirms that the choices prisoners make that determine their caste position or status in prisoner society are interwoven with their ethnic and religious identity, whether this is self-ascribed or constructed for them. For the Uzbeks in Farhod’s colony, placement in a ‘red barrack’ - that is, one for the activist prisoners - was considered shameful and reflected badly on the whole Uzbek community. As a result, substantial efforts were made to dissuade newly arrived prisoners from becoming activists, even though cooperating with the administration could benefit them personally, such as in their application for early release and access to enhanced privileges. To counter the temptations to align with the administration, Uzbek prisoners went out of their way to provide material support and mentoring to new arrivals; a member of the Uzbek community would be assigned to visit fellow Uzbek nationals fresh off the prison transport during their two-week period of quarantine. The visit, Farhod explained, allows the Uzbek to determine if a newcomer is a ‘decent person’ ( *poryadochnii muzhik*) and if deemed to be so, whether he needed support with basics, such as food, tea, a pack of cigarettes, or sanitary items (soap, a dental kit). During the quarantine period, the unofficial mentor would explain the formal and informal rules of a particular zone and convince the new prisoner of the importance of maintaining distance from the administration.

At a more general level, taking care of compatriots or, in some cases, co-regionalists ( *zemlayki*) in need of material support is understood as a question of honour and reputation among transnational prisoners, and failure to do so, a source of shame. Mutual assistance to Uzbeks who, for example, are not supported by parcels from outside the colony can be supported by ‘the box’ ( *korobka*), a mutual aid fund to which every Uzbek, who is able, must contribute. For example, if someone received a parcel containing tea, cigarettes, fruits, bread, canned meat, or other produce from family, friends, or other close people outside the colony, there was an expectation that a portion would be given up to the *korobka*. Uzbeks without external support would be expected to work in the production zone and pay a part of their monthly earnings to the box. In this way, a social safety net existed exclusively for Uzbeks, which simultaneously maintained their dignity and honour of the community at large. This mutual aid practice is comparable to typical *mahalla*-based social safety and risk-stretching practices that are part and parcel of everyday life in Uzbekistan. Such ethnicity-driven mutual aid practices also existed among other tight-knit ethnic groups in the zone, such as Tajiks, Chechens and Dagestanis. It is tempting to draw parallels between the Uzbek system of mutual support with the Thieves-in-Law’s communal fund but the two systems differ fundamentally. While latter-day thieves-in-law will insist that the *obshak* provides a safety net for less fortunate sub-culture members, in practice the first call on it is support for the comfortable lifestyle of the criminal leaders. Furthermore, access to the *obshak* depends upon an individual prisoner’s status as determined by the thieves-in-law. Supporters of the traditional prisoner sub-culture like to lay claim to moral superiority of the *ponyatie* over formal law, but such claims are, to say the least, tenuous. ^71^ In contrast, the *korobka* is informed by and compatible with Sharia law. Furthermore, the organizing principle of mutual support among Uzbek transnational prisoners is ethnicity and *zemlyachestvo*, which is not the case with today’s criminal sub-cultures. In this regard, *obshak* and *korobka* are not same given that the former functions based on the economic logic and power geometries of the traditional criminal subculture, while *korobka* emerged as a risk-stretching strategy among religiously (Muslims) and ethnically homogenous groups.

### Contact with the outside world

An essential component of the informal system of governance in Russian correctional colonies is the shadow market, usually run by the criminal sub-culture, but in which all prisoners can participate. The primary goods circulating in the shadow market are mobile phones and drugs, which are imported into colonies by illegal channels. The currency for exchange is money - earned in zone work, monetary transfers, or card games - and legitimately received trade goods such as cigarettes and tea received from outside. In participating in the purchase and sale, especially of illegal goods circulating in the shadow market, Uzbek prisoners automatically put themselves at risk of being identified as ‘regime violators,’ which carries penalties including periods in the colony’s disciplinary cells and loss of privileges. Therefore, prisoners must exercise caution to avoid discovering the illegal items during searches. Even in a black colony like Farhod’s, prisoners confine their use of cell phones to after lights out and hide forbidden items in secret hiding places ( *gashniki*). 

Using a mobile phone is essential for transnational prisoners to maintain contact with their relatives back in the Fergana valley and the communities of the migrants in large Russian cities, which can fulfil a variety of functions for their imprisoned compatriots. These include sending produce parcels and appealing to authority figures in the Muslim community beyond the prison walls to adjudicate conflicts with other Muslim prisoners. ^72^ Poor access, time limits, and monitoring of calls deter transnational prisoners from relying on the payphones installed in colonies. For this reason, cell phones command a high price in the shadow market. ^73^

Participating in the shadow market in the zone was not the only illegal activity in which the Uzbek prisoners took part in Farhod’s zone; they also played cards ( *qimor*) at night organized and policed by the criminal sub-culture. But these relative freedoms depended upon the handover of power by the administration to the colony’s criminal subculture. Many of the activities that Farhod describes about the everyday life of Muslim prisoners in his zone, in red and regime colonies is subject to disciplinary censure. In their negotiations with prison administrations, the degree of latitude prisoners have should not be overstated, even in black colonies. This was amply demonstrated during the COVID-19 pandemic when prison lockdowns seriously disrupted prisoners’ communication and supply networks with the outside world in all categories of facilities. These bans extended to prohibiting prisoners receiving parcels from, and communicating by letter and telephone with, the outside world. It will be a long time before the true story of COVID-19 in Russia’s prisons is told and, indeed, the truth may never be known. ^74^ However, it is reasonable to assume that Muslim prisoners from Central Asia and the Caucasus were adversely affected in particular and discriminatory ways by the restrictions on communication with their migrant networks and relatives. The interruption of the daily flow of information, money, electronic devices, halal meat, food, and produce parcels will have dramatically changed many facets of the lives of Tajik and Uzbek Muslim prisoners. Muslim prisoners must have suffered disproportionately from these restrictions because of the heavy reliance on produce parcels to meet their partial dietary requirements. Their ability to practice religion in groups and socializing and fast-breaking events will also have been affected during the Ramadan period.

The covid-19 pandemic revealed the extent to which the Russian Prison service was able to ignore its obligation under the European Convention of Human Rights to treat people equally regardless of their religious beliefs, race, and ethnicity. It brought home the degree to which transnational Muslim prisoners imprisoned thousands of miles from home, have had to find ways of self-provisioning that have included having to rely on negotiation with criminal power hierarchies or indulge in rule-breaking to be able to practice their basic ethno-religious and ethno-cultural customs.

## Conclusion

The arrival of large numbers of foreign migrant prisoners in the past two-three decades has introduced a new dimension into the culturally diverse Russian prison. Prison society has begun to be shaped in ways that undermine its previous class and criminal offense-based group formation and stratification. True, there have been previous moments in Soviet history when groups based on national and religious affinity have surfaced in prisons, for example, at times of crisis and conflict but the new groupings of transnational prisoners from Central Asia are different. Group formation among transnational prisoners can be viewed as a spontaneous, adaptive response to the precarious situation Muslim migrants occupy on both sides of the colony fences, but the evidence of people like Farhod of the shared rituals and patron-client relationship with indigenous Russian Muslims from the North Caucasus also suggest that Muslim subcultures have become a more-or-less institutionalized feature of Russian prisons, today. The Chechen *polozhenets*, who enforced the thieves law in the zone’s everyday life, also informally protected the interests of Muslims in the zone, even though his actions contradicted his role as a local representative of the *vory*. One possible inference is that the Muslim sub-culture has emerged as a horizontally-organised group revolving around the Sharia law and Muslim communal values of solidarity and mutual support, newly-emerging intermediate groupings which operate vis-à-vis the traditional criminal subculture. The result is the emergence of a far more complicated and plural power geometry between the prison administration and prisoners and between Russian traditional criminal sub-culture and transnational Muslim prisoners.

 These developments have taken place against the backdrop of significant transformations in Russian society, including the rise of xenophobia and, as is obvious in the war in Ukraine, the elevation of ethno-religious notions of national belonging. It is not surprising that these processes have filtered into prisons aided by the communal living of the majority of prisoners and their ability to forge links with the outside world thanks to a combination of modern communication technologies, reforms supporting prisoners’ contacts with the outside world, and extraordinary levels of corruption among prisoner personnel who turn a blind eye to or participate in the penal shadow economy. Having to deal with large groups of Muslim prisoners with overlapping networks defined by ethnicity, religion, and individual lifestyle choices is a new challenge for FSIN. To date, it has approached this challenge with the familiar combination of carrot and stick, building mosques in colonies with large numbers of Muslims and hiring their own imams to lead Friday prayers but, simultaneously, cracking down on too enthusiastic manifestations of religious belief. The confusion that FSIN faces is illustrated by the situation in Farhod’s colony, where we learned that the administration recruited the help of a Chechen, who happened to be the leader of the prison sub-culture, to suppress other Muslims whom the official internal surveillance system logged as followers of the ‘wrong’ sort of Islam. There are many possible explanations for the motivations of the various parties in this story. The administration, for example, may, indeed, have had evidence that the Dagestani Muslims were forming a terrorist cell and so moved to nip it in the bud. More likely, however, was that it needed to provide superiors with evidence of the success of its ‘campaign against extremism’ or, even more likely, that it was protecting its own access to the shadow economy operated by the sub-culture. The possible motivation of the Muslim parties, whether understood as ethnic, religious or criminal-authority actors, is similarly multifaceted. But the dominant message from the research with Central Asian transnational Muslim prisoners, is the need to revise the prevailing understanding about the power dynamics in penal space in Russian penal institutions to take account of the ‘end of cosmopolitan penal space’ if such ever really existed in the USSR and recognize them today as sites of ethnic and religious pluralism.

## Data Availability

The restrictions on the data collection, processing, and transfer are laid down in the Data Management Plan for project no. 788448 and the Data Management and Ethics Process Plan submitted and approved by the ERC Executive Committee and the Helsinki University Ethics Committee and is subject to periodic monitoring by the project's Ethics Advisory Board. The interview data used in the article consist of transcripts of conversations with research participants who had served custodial sentences in Russian prison institutions for criminal offences, and who were then returned after the end of their sentence to Uzbekistan. The interviews were taken by one team member in Uzbekistan. The transcribed data were deposited in the shared space of the gulagechoes project and access restricted to the team members, with their use in an article subject to approval by the two authors of this paper. The interviews have recently been deposited in Zenodo (Judith Pallot. (2023) “Uzbekistan sample_Bita_Male.” Uzbekistan Interview Transcripts. Zenodo. https://doi.org/10.5281/zenodo.7924716). They are under embargo until August 2030. This is as per the requirements of the ERC EC and Helsinki Ethics Committee. The project's independent Ethics Advisory Board that the ERCEA required the project to set up has at its meeting discussed the question of open access to the data in relation to the evolving political situation in Uzbekistan but have seen no reason to apply for permission to the institutional bodies to transfer the data to restricted access status. However, before the end of the project in August 2024, we will discuss which corpuses of the embargoed data we might be able to transfer to restricted or fully open access but this is unlikely to apply to any of the Uzbek interviews. For data queries please contact the corresponding author at judith.pallot@helsinki.fi.

## Footnotes

^1^ According the International Migration Organisation 2019 report, more than 11 million foreign-born individuals reside within the territory of Russian Federation: https://governingbodies.iom.int/system/files/en/council/110/Statements/Russian%20Federation%20-%20Statement%20-%20110th%20Council.pdf accessed 18 ^th^ May 2023. Undocumented migrants are not included in these official statistics. No consensus exists among migration scholars and experts regarding the number of foreigners/migrants residing in Russia. Estimates vary, with the number of migrants living in Russia put at somewhere between 9 and 18 million individuals depending on the source used.

^2^ https://kun.uz/en/news/2023/02/22/uzbekistan-again-becomes-main-source-of-labor-migrants-for-russia (accessed 13/05/2023)

^3^ Abashin, S., 2014. Migration from Central Asia to Russia in the New Model of World Order. *Russian Politics & Law*, 52 (6), 8–23.

^4^ Schenk, C., 2018. *Why Control Immigration? Strategic Uses of Migration Management in Russia.* Toronto, ON: University of Toronto Press.

^5^ Urinboyev, R., 2020. *Migration and Hybrid Political Regimes: Navigating the Legal Landscape in Russia*. Oakland, CA: University of California Press.

^6^ TASS, 2021. Zamglavy MVD Rossii napomnil nelegal’nym migrantam iz stran SNG o groziashei im deportasii. *TASS*. Available from: https://tass.ru/obschestvo/11169343 accessed 13/05/2023

^7^ Kuznetsova, I. and Round, J., 2018. Postcolonial migrations in Russia: the racism, informality and discrimination nexus. *International Journal of Sociology and Social Policy*.

^8^ Eraliev, S. and Urinboyev, R., 2020. Precarious Times for Central Asian Migrants in Russia. *Current History*, 119 (819), 258–263.

^9^ Pakes, Francis, and Katrine Holt. "Crimmigration and the prison: Comparing trends in prison policy and practice in England & Wales and Norway." *European Journal of Criminology* 14, no. 1 (2017): 63–77.

^10^ Wacquant, Loic. "Suitable enemies' foreigners and immigrants in the prisons of Europe." *Punishment & society* 1, no. 2 (1999): 215–222. On the same point see also, Ugelvik, Thomas. "The incarceration of foreigners in European prisons." In *The Routledge handbook on crime and international migration*, pp. 107-120. Routledge, 2017.

^11^ Ugelvik, Thomas, and Dorina Damsa. "The pains of crimmigration imprisonment: Perspectives from a Norwegian all-foreign prison." *The British Journal of Criminology* 58, no. 5 (2018): 1025–1043.

^12^ Moore, J.M., 2015. The ‘New Punitiveness in the context of British imperial history. *Criminal Justice Matters*, *101*(1), pp.10–13; De Giorgi, A., 2010. Immigration control, post-Fordism, and less eligibility: A materialist critique of the criminalization of immigration across Europe. *Punishment & Society*, *12*(2), pp.147–167

^13^ The colony is described in on-line news articles at https://zona.media/article/2017/06/15/dubravlag and https://bk55.ru/news/article/148392/ (both accessed 13/05/2023). According to Interfax.ru statistics for 2019 the number of foreign prisoners from the far abroad in Russian facilities was 350, the majority from south-east Asia.

^14^ Under Article 12 of the criminal correction code of the Russian Federation, foreign nationals have certain different rights with respect to the language of communication and correspondence, and a right to consular visits. Whilst they are held in similar conditions as Russians and should be treated the same, prison NGOs report that they are subject to discriminatory treament and verbal abuse by personnel and other prisoners alike.

^15^ We know, for example, that Russia failed to inform the Council of Europe of the number of foreign prisoners incarcerated in its prisons: https://www.bbc.com/russian/news-56674300 accessed 5/12/2023

^16^ https://fergana.agency/articles/110276/ accessed 5/12/2023

^17^ For 2015, 2016 and 2017 statistics, https://russian.rt.com/russia/article/411991-fsin-tyurmy-inostrancy. 2018 statistics: https://russian.rt.com/russia/news/567669-inostrancy-rossiya-tyurmy-umenshenie 2019 statistics https://vz.ru/question/2020/7/31/1052613.html 2020 statistics: https://ria.ru/20130304/925376615.html all accessed 15th July 2023

^18^ For 2015, 2016 and 2017 statistics, https://russian.rt.com/russia/article/411991-fsin-tyurmy-inostrancy. 2018 statistics: https://russian.rt.com/russia/news/567669-inostrancy-rossiya-tyurmy-umenshenie 2019 statistics https://vz.ru/question/2020/7/31/1052613.html 2020 statistics: https://ria.ru/20130304/925376615.html When the authors consulted these sites, they were accessible. After the Russian invasion of Ukraine of 24th February 2022, the sites of the TV channel RT and of the official government news agency RIA Novosti were blocked for international access. They are, however, still accessible using VPN.

^19^ RIA Novosti, 2020. FSIN: kazhdyi tretiy osuzhdennyi inostranes v Rossii sidit za narkotiki. Available from: https://ria.ru/20130304/925376615.html When the authors consulted this site, it was accessible. After the Russsian invasion of Ukraine of 24th February 2022, the ria.ru site was blocked for international access but it is accessible using VPN.

^20^ https://nuz.uz/o-migracii/43451-skolko-prestupleniy-prihoditsya-v-rossii-na-dolyu-priezzhih-iz-centralnoy-azii.html & https://uzmetronom.agency/2020/05/25/na-rodine-i-balandjor-vospitatel.html both accessed 13/05/2023

^21^ RT, 2018. FSIN: chislo inostrannyh zakliuchennyh v rossiyskih tiur’mah sokratilos’ pochti na 6%. Available from: https://russian.rt.com/russia/news/567669-inostrancy-rossiya-tyurmy-umenshenie When the authors consulted this site, it was accessible. After the Russsian invasion of Ukraine of 24th February 2022, the rt.com site was blocked for international access but it is accessible using VPN.

^22^ https://www.migrationwatchuk.org/briefing-paper/484/foreign-national-offenders for 2019 and for 2021 https://researchbriefings.files.parliament.uk/documents/SN04334/SN04334.pdf for USA 2022 includes no citizenship https://www.bop.gov/about/statistics/statistics_inmate_citizenship.jsp all accessed 13/05/2023

^23^ Martirosyan, K. K., Muratov, R. Kh., & Trofimova, O. A. (2017). Terrorizm s ispol'zovaniem seti Internet, in *Pravovoe regulirovanie sovremennogo obshchestva: teoriya, metodologiya, praktika,* 433–435; Curro, Costanza, Pallot, Judith and Zeveleva, Olga, (2023), “Ethnicity in Soviet and post-Soviet Prisons” in *The Oxford Research Encyclopedia on Race, Ethnicity and Communication*, London and New York: Oxford University Press.

^24^ For a discussion of what is meant by traditional Islam see, Di Puppo, L. and Schmoller, J., 2020. Introduction: Studying Islam from below and interrogating divisions in contemporary Russia. *Contemporary Islam*, *14*, pp.99–110.

^25^ The Russian Prison service has a number of Academies - higher education institutions - in which prison officers receive their training. Courses last five years, the *kursanti* passing out with military rank. These institutions have their own journals in which prison service staff and researchers can publish articles based on prison research. Articles on prisons are also published in journals of Ministry of Justice journals. There is little prison research conducted in the Ministry of Education higher education institutions.

^26^ Getty, J. Arch, Gabor T. Rittersporn, and Viktor N. Zemskov, 2018 "Victims of the Soviet penal system in the pre-war years: a first approach on the basis of archival evidence." In *The Soviet Union*, Routledge, : 153–185. This was published first in the American Historical Review; 1993, (98/4) p. 1017–1049

^27^ Zemskov, V.N, 1991, GULAG (istoriko-sotsiologicheskii aspect), *Sotsiologicheskie issledovaniya* : 10–27

^28^ The GULAGECHOES project collected interviews with Muslim Russian nationals in various regions of the Russian Federation. These are currently being analysed and prepared for publication.

^29^ Op cit., Martirosyan et al, 2017.

^30^ A longer discussion of the analysis of the FSIN researchers of the radicalisation ‘problem’, its causes and consequences by one of the authors is to be found in the section of Muslim prisoners in Curro, Costanza, Pallot, Judith and Zeveleva, Olga, (2022, November 22), “Ethnicity in Soviet and post-Soviet Prisons” in *The Oxford Research Encyclopedia on Race, Ethnicity and Communication*, London and New York: Oxford University Press’ https://doi.org/10.1093/acrefore/9780190228613.013.1328.

^31^ Maslov, I. S. (2019). Deyatel'nost' totalitarnykh sekt v penitentsiarnoi sisteme rossiiskoi federatsii-ugoroza natsional’noi bezopasnosti rossiiskoi federatsii. In *Aspirantskie tetradi*, 86–90: p.88

^32^ Kopylov, V. V., & Vanteeva, A. Yu. (2019). Realizaciya prava zaklyuchennykh, nakhodyashchikhsya v mestakh lisheniya svobody federal' noii sluzhby ispolneniya nakazaniya Rossii, na svobodu veroispovedovaniya. *Vestnik Moskovskogo universiteta MVD Rossii*, (4), 29–32: 31–2

^33^ The law is from 1 Jun 2020 Article 53.1 of the Criminal Code of the RF. This law is discussed by Judith Pallot, at https://blogs.helsinki.fi/gulagechoes/2021/08/19/what-lies-behind-fsins-promotin-of-forced-labour-used-as-an-alternative-to-deprivation-of-liberty2/ and the precursor use of forced labour by BAMLAG by Mikhail Nakonechnyi at https://blogs.helsinki.fi/gulagechoes/2021/06/03/bamlags-lingering-shadow/ both accessed 13/05/2023

^34^ For a summary of western approaches see, Silke, A., ed 2014, Prisons, Terrorism and Extremism, Routledge, and Quraishi, Muzammil 2008, "Researching Muslim prisoners." *International Journal of Social Research Methodology* 11, no. 5: 453–467.

^35^ There are very few empirical studies examining how Muslims in western jurisdictions experience imprisonment that is on a par with the extensive literature on Black and Hispanic prisoners in the USA or Maori in Australia. The studies we do have tend to focus primarily upon the evaluation of various preventative interventions to steer prisoners away from a path leading towards violent radicalisation such as, Khosrokhavar, Farhad 2013, "Radicalization in prison: The French case." *Politics, Religion & Ideology* 14, no. 2: 284–306; Beckford, James A. 2005, "Muslims in the prisons of Britain and France." *Journal of Contemporary European Studies* 13, no. 3: 287–297. Beckford, James, Daniele Joly, and Farhad Khosrokhavar. 2016, *Muslims in prison: Challenge and change in Britain and France*. Springer. There are few studies of the everyday experiences of Muslim prisoners in western prisons, a notable exception being Phillips, Coretta, 2012, *The multicultural prison: Ethnicity, masculinity, and social relations among prisoners*. Oxford University Press. One source on radical Islam in Russian prisons is Sweet, Julia. "From the Post-Soviet Godless Legacy to Radical Islam: Russian Converts." *Global Security Studies* 7, no. 1 (2016).

^36^ Yavorskii, M. A. 2016, Prichiny radikalizatsii osuzhdennykh v mestakh lisheniya svobody v Rossii i za rubezhem. In *Natsional'naya bezopasnost': strategicheskie prioritety i sistema obespecheniya: Materialy Mezhdunarodnoi nauchno-prakticheskoi konferentsii*: 166–173

^37^ Pallot, Judith, Laura Piacentini, and Dominique Moran, 2010, "Patriotic discourses in Russia's penal peripheries: Remembering the Mordovan Gulag." *Europe-Asia Studies* 62, no. 1: 1–33.

^38^ On the identification of push-and-pull factors of migration: Denisenko, M. and Varshavskaya, Y., 2013. *Migrants at the Russian Labour Market: Characteristics, Status, Mobility*. Moscow: National Research University - Higher School of Economics, No. WP3/21 SEARCH Working Paper); on the difficult living and working conditions among migrants, Kuznetsova, I. and Round, J., 2018. Postcolonial migrations in Russia: the racism, informality and discrimination nexus. *International Journal of Sociology and Social Policy*); on the impact of immigration laws and practices Kubal, A., 2019. *Immigration and Refugee Law in Russia*. Cambridge: Cambridge University Press; on racism, xenophobia, and the exclusion of migrants Agadjanian, V., Menjívar, C., and Zotova, N., 2017. Legality, Racialization, and Immigrants’ Experience of Ethnoracial Harassment in Russia. *Social Problems*, 64 (4), 558–576; on corruption and migration governance, Schenk, C., 2021. Producing state capacity through corruption: the case of immigration control in Russia. *Post-Soviet Affairs*, 1–15); on the political economy of housing and migrants’ everyday lives, Reeves, M., 2016. Diplomat, Landlord, Con-artist, Thief: Housing Brokers and the Mediation of Risk in Migrant Moscow. *The Cambridge Journal of Anthropology*, 34 (2), 93–109; on female migrants’ experiences, Eraliev, S. and Heusala, A.-L., 2021. Central Asian female migrants’ transnational social spaces: Straddling illegality and tradition. *In*: R. Turaeva and R. Urinboyev, eds. *Labor, Mobility and Informal Practices in Russia, Central Asia and Eastern Europe: Power, Institutions and Mobile Actors in Transnational Space*. London: Routledge, 114–132); on migrant illegality and the shadow economy, Urinboyev, R. and Eraliev, S. (2022) *The Political Economy of Non-Western Migration Regimes*. Cham: Palgrave Macmillan; on migrant religiosity and the role of Islam in migrants’ everyday lives (Turaeva, R., 2018. Imagined Mosque communities in Russia: Central Asian migrants in Moscow. *Asian Ethnicity*, 1–17.; ton he radicalization of migrants, Poletaev, D., 2019. From mistrust to solidarity or more mistrust? Russia’s migration experience in the international context. *Russia in Global Affairs*, 17 (1), 171–197; on transnational practices and the livelihoods of migrants, Urinboyev, R., 2021. Smartphone Transnationalism in Non-Western Migration Regimes: Transnational Ethnography of Uzbek Migrant Workers in Russia. *In*: R. Turaeva and R. Urinboyev, eds. *Labor, Mobility and Informal Practices in Russia, Central Asia and Eastern Europe: Power, Institutions and Mobile Actors in Transnational Space*. London: Routledge, 89–113; and on family migration (Nikiforova, E. and Brednikova, O., 2018. On labour migration to Russia: Central Asian migrants and migrant families in the matrix of Russia’s bordering policies. *Political Geography*.

^39^ The prison service academies are higher education institutions subordinate to the Prison Service in which prison officers receive their training. They were set up originally by the NKVD and have continued on to the present day, albeit with changed syllabuses. Courses last five years, the *kursanti* passing out with military rank. These institutions have their own journals in which prisons service staff and researchers can publish articles based on prison research. Articles on prisons are also published in journals of Ministry of Justice journals. There is little prison research conducted in Ministry of Education higher education institutions.

^40^ This search was conducted as part of a Leverhulme Foundation Trust (UK) grant EM-2018–21, “Ethic identity construction among Muslim prisoners in the USSR and Russia” held by Professor Pallot 2019–2020.. The search was conducted by Research Assistant, Dr Elena Katz, and included in an unpublished report on the project.

^41^ Laruelle, M.,2007. Xenophobia and Its Political Use in Russia: The Skinhead Example. *Revue internationale et strategique*, No 68 (4), 111–119; Laruelle, M., 2017. Is Nationalism a Force for Change in Russia? *Daedalus*, 146 (2), 89–100.

^42^ Ozodlik Radiosi/Radio Free Europe Uzbek Service, 2018. “Novaya Gazeta” *Maqolasidan so’ng qamoqdagi qiynoq ustidan ish ochildi* (After the "Novaya Gazeta" article, a case was opened on torture in prison). Available in the Uzbek language from: https://www.ozodlik.org/a/29385228.html accessed 3rd July 2023.

^43^ Ozodlik Radiosi, 2016. Arxangelsk qamog‘idagi o‘zbek tutqunini ham pasport o‘ylantirmoqda. Available from: https://www.youtube.com/watch?v=eTxHlj4pM0g accessed 14/05/2023; Ozodlik Radiosi, 2018. Rossiya *qamog‘idagi O‘zbek muhojirlari ochlik elon qildi*. Available from: https://fb.watch/7YxaSNF7qi/ accessed 14/05/2023 *Ozodlik Radiosi, 2019. Rossiya qamog‘idagi o‘zbek mahbusi o‘zining tahqirlanaëtganini iddao qildi* (An Uzbek prisoner in Russian custody claimed that he was being abused), available from: https://www.ozodlik.org/a/30316278.html accessed 14/05/2023

^44^ Peshkov, I., 2015. The Communal Apartment Under ‘Special Surveillance’: The Legacy of the Soviet Gulag in Multiethnic Criminal Subcultures in Eastern Siberian Prison Camps. *Laboratorium-Russian Review of Social Research*, 7 (1), 71–91.

^45^ Ibid.p.86-7

^46^ Anisimkov, V, Rossiya v Zerkale ugolovvnykh traditsii tyurmy, (2017), *Litres*. Antonian, Iu. M. and Kolyshshnitsyna, E. N., *Motivatisia povedeniia osuzhdennykh: monografiia* (Motivations for prisoners; a monograph) (Unity: Moscow, 2009); Butler, Michelle, Gavin Slade, and Camila Nunes Dias. "Self-governing prisons: Prison gangs in an international perspective." *Trends in Organized Crime* (2018): 1–16; Oleinik, Anton N., Alain Touraine, and Sheryl Curtis. *Organized crime, prison and post-Soviet societies*. Routledge, 2017; Varese, Federico. *The Russian Mafia: private protection in a new market economy*. OUP Oxford, 2001; Varese, Federico, Jakub Lonsky, and Yuriy Podvysotskiy. "The resilience of the Russian mafia: an empirical study." *The British Journal of Criminology* 61, no. 1 (2021): 143–166; GGG, Olienik, Varese, Slade, Kyrgyz article etc.

^47^ For the full range of views on the vory-v-zakony see the following Oleinik, A. N. "Prison subculture in Russia: from everyday life to state power." *Moscow, Infra-M* (2001), Siegel, Dina. "Vory v Zakone: Russian organized crime." In *Traditional organized crime in the modern world*, pp. 27–47. Springer, Boston, MA, 2012; Slade, Gavin. "The threat of the thief: Who has normative influence in Georgian society? Dispatches." *Global crime* 8, no. 2 (2007): 172–179; Varese, Federico, Jakub Lonsky, and Yuriy Podvysotskiy. "The resilience of the Russian mafia: an empirical study." *The British Journal of Criminology* 61, no. 1 (2021): 143–166.

^48^ Oleinik, Anton N., Alain Touraine, and Sheryl Curtis. *Organized crime, prison and post-Soviet societies*. Routledge, 2017; Pallot, Judith and Laura Piacentini,. *Gender, geography, and punishment: The experience of women in carceral Russia*. Oxford University Press, 2012; Pallot, Judith. "The Gulag as the crucible of Russia's 21st-century system of punishment." *Kritika: Explorations in Russian and Eurasian History* 16, no. 3 (2015): 681–710; Piacentini, Laura, and Judith Pallot. "‘In Exile Imprisonment’in Russia." *British Journal of Criminology* 54, no. 1 (2014): 20–37; Piacentini, Laura, and Gavin Slade. "Architecture and attachment: Carceral collectivism and the problem of prison reform in Russia and Georgia." *Theoretical Criminology* 19, no. 2 (2015): 179–197

^49^ A longer discussion of Soviet and Russian penal architecture is to be found in Pallot, J. and Zeveleva, O., 2022. The Architecture and Design of the Communist and Post-Communist Prison in Europe. In *The Palgrave Handbook of Prison Design*: 227–260;; Piacentini, L. and Slade, G., 2015. Architecture and attachment: Carceral collectivism and the problem of prison reform in Russia and Georgia. *Theoretical Criminology*, *19*(2), pp.179–197.

^50^ Pallot. J. 2012 “Socialism in one barrack”, in Pallot J and L Piacentini, *Gender Geography and Punishment; Women’s experiences of carceral Russia*: OUP: 96–119. For a discussion of prisoner self-government in Ukrainian correctional colonies which, as in Russia, have perpetuated the Soviet-era prison design see, Symkovych, A., 2018. The Ukrainian response to Sykes: prisoner hierarchy and self-rule—power, legitimacy, and dynamics. *The British journal of criminology*, *58*(5), pp.1087–1106.

^51^ For a discussion of the varied roles of the prison officer forthcoming 2023, Pallot, Judith, The Prison Officer in Post-Soviet Russia, in Eds: Arnold Helen, Matthew Maycock and Rosemary Ricciardelli, *Prison Officers: International Perspectives on Prison Work*, Palgrave Macmillan.

^52^ In fact, ‘zona’ is a generic term that when applied in the penal context, can refer to a space of confinement at a variety of spatial scales. It is usually used in the singular form. As well as being used to refer to a single penal institution such as a sizo (remand prison) or correctional colony, it also applies to a cluster of penal institutions subordinated to a regional prison authority (UFSIN) or part of the territory within a penal institution. In some post-Soviet countries zona refers to the old Soviet-era type facilities used to draw a contrast with the ‘Europrisons’, or any facility with cellular, rather than communal accommodation.

^53^ These designations do not refer to women’s correctional colonies which are said to be universally ‘red’, nor are they applicable to the very highest security facilities such as prisons (t *yurmy*) and special regime colonies including those for ‘lifers’.

^54^ Butler, M., Slade, G. and Dias, C.N., 2018. Self-governing prisons: Prison gangs in an international perspective. *Trends in Organized Crime*, pp.1–16.

^55^ Oleinik, Anton N., Alain Touraine, and Sheryl Curtis. 2017, *Organized crime, prison and post-Soviet societies*. Routledge, 2017

^56^ The term orange derives from its original attachment to Georgian and Azerbajani *vory-v-zakony* who agreed to negotiate a distribution of power with prison authorities (oranges because this was the product both republics were known for supplying to the rest of the USSR). Later the term came to be applied to prisoners who paid to be crowned as a *Vor*. Its use is perjorative when used by *Vor* purists who regret the passing of the strict interpretation of the ‘understanding’ which prohibits any cooperation with the administration.

^57^ Whereas during the Soviet period the penal economy was strong enough for there to be work for all prisoners of working age and fitness to work. In post-Soviet Russia there has been sufficient work for only about 30% of the prison population to work, although this is now rising. Prisoners’ motivations for working vary - to earn money, to counteract boredom, to avoid punishment for refusing to work, or to avoid placement in a ‘black’ barrack.

^58^ Prior to the end of the first decade of the millenium it was possible for non-FSIN insiders and independent researchers to gain access for the purpose of interviews, observation and surveys to FSIN facilities. For foreigners access was withdrawn earlier, in 2007, when a team of UK researchers who had joint project with a prison service Academy had their visas cancelled; see chapter 2 of Pallot, Judith, Laura Piacentini, and Dominique Moran. *Gender, geography, and punishment: The experience of women in carceral Russia*. Oxford University Press, 2012.

^59^ Approval of the ERC Executive Agency Ethics Committee was required before the transfer of funding for the GULAGECHOES project to the University of Helsinki, which included the approval of the beneficiary’s Ethics Committee and the production of a Data Management Plan consistent both with 2018 EU GDPR and the relevant Uzbek laws covering, in particular, the conditions for the transfer of personal data to EU from a non-EU country.

^60^ Reyes, V., 2020. Ethnographic toolkit: Strategic positionality and researchers’ visible and invisible tools in field research. *Ethnography*, 21 (2), 220–240.

^61^ On the source of power imbalances in interviews, see, Briggs, C.L., 2002. Interviewing, power/knowledge, and Social inequality. *Handbook of interview research: Context and method*, pp. 910–922; Anyan, F., 2013. The Influence of Power Shifts in Data Collection and Analysis Stages: A Focus on Qualitative Research Interview. *Qualitative Report*, *18*, p.36; Ross, K., 2017, July, Making empowering choices: How methodology matters for empowering research participants. In Forum Qualitative Sozialforschung/Forum: Qualitative Social Research (Vol. 18, No. 3); Rubin, H.J. and Rubin, I.S., 2011. Qualitative interviewing: The art of hearing data. Sage.

^62^ For the methodological issues surrounding interviewing prisoners see, Earle, R., 2014. Insider and out: Making sense of a prison experience and a research experience. *Qualitative Inquiry*, *20*(4), pp.429–438; Newman, D. J., 1958, Research Interviewing in Prison, 49 *Journal of Criminal Law and Criminology* 49 (2) pp. 127–132; and on using prison argot in prison interviews, Pallot, J. (2022). I was a Special Operations Officer in a Russian Correctional Colony, but I’ll Speak Georgian in The Politics of Language in Qualitative Research in the Former Soviet Union, in C. Bading,J. Wintzer &K. Kazzazi eds.(Foreign) Language and Qualitative Social Research: Research Strategies in Intercultural Contexts, Springer Spectrum,(in press 2022). In *Language and Qualitative Social Research: Research Strategies in Intercultural Contexts, Springer Spectrum,(in press 2022)*. Springer

^63^ Hoffmann, E.A., 2007. Open-ended interviews, power, and emotional labor. *Journal of contemporary ethnography*, 36 (3), 318–346.

^64^ The ground plan of correctional colonies, which dates back to the reforms of 1960, is described in Pallot, J., Zeveleva, O. (2023). The Architecture and Design of the Communist and Post-Communist Prison in Europe. In: Moran, D., Jewkes, Y., Blount-Hill, KL., St. John, V. (eds) The Palgrave Handbook of Prison Design. Palgrave Studies in Prisons and Penology. Palgrave Macmillan, Cham. https://doi.org/10.1007/978-3-031-11972-9_9 accessed 14/05/2023

^65^ This was a potential far-reaching reform that would have changed the physical and management structure of the system inherited from the USSR, but over time the most radical proposal that would have eliminated correctional colonies and replaced them by institutions with cellular accommodation was whittled away. *Kontseptsia razvitiya ugolovno-ispolnitel’noi sistemy v Rossiiskoi Federatisii do 2020* (Conception for the Development of the Russian Prison Service to the year 2020). The original has disappeared from the Russian government website but it can be accessed on a DNR prison service website at http://ukrprison.org.ua/articles/1264936781 accessed 14/05/2023

^66^ There are red colonies, for example, where the timetabling of morning exercises or head counts coincide with morning prayers and no modifications to mealtimes made during Ramadan.

^67^ These are (1) the profession of faith; (2) praying five times a day; (3) giving *zakat* (alms), i.e., donating a certain portion of one's wealth; (4) fasting during Ramadan; and (5) making a hajj (pilgrimage) to Mecca in Saudi Arabia.

^68^ The criminal corrections code of the Russian Federation (Code on the execution of Senetnces) of 18 december 1996, as amended up to J19 June 20: https://www.ilo.org/dyn/natlex/natlex4.detail?p_isn=71645&p_lang=en accessed 3 ^rd^ July 2023

^69^ The variability was confirmed in one other interview taken with indigenous Russian Muslims in other field work sites for the GULAGECHOES project which have yet to be published.

^70^ The claim is that (with which they associated Uzbeks, Kyrgyzs, Kazakhs, Tatars, Azerbaijanis) came from the Urals and Siberia and colonized the Central Asia, a region that was originally inhabited by Persian-speaking groups. Tajiks, as Farhod said, also claimed that Samarkand and Bukhara were originally Tajik cities which have nothing to do with contemporary Uzbekistan.

^71^ Pallot, Judith, and Elena Katz. *Waiting at the prison gate: Women, identity and the Russian penal system*. Bloomsbury Publishing, 2016. Chapter 4.

^72^ https://blogs.helsinki.fi/gulagechoes/2020/06/17/muslim-prisoners-in-russia-during-the-the-covid-19-pandemic/ accessed 24 ^th^ May 2023

^73^ For example, if a basic Nokia mobile phone cost 5000 roubles (US$125) in a store, a *baryga* sold it to prisoners for 15 000 roubles (US$230). Smartphones were even more expensive. If the cheapest Chinese Huawei smartphone costs 13 000 roubles (US$200) outside prison, it costs 30 000 roubles (US$450) inside. The price of an iPhone could reach up to 150 000 roubles (US$2500).

^74^ On the history of the secrecy surrounding epidemics in Imperial Russia, Soviet and post -Soviet prisons see, Nakonenchnyi, Mikhail and Judith Pallot, (forthcoming 2023), “Silences and omissions in reporting epidemics in Russian and Soviet Prisons, 1890–2021”, *The Oxford Journal of the History of Medicine.*
