# Supplementary material for: Ethnic and religious identities in Russian penal institutions: A case study of Uzbek Transnational Muslim prisoners
Source: Open Res Eur. 2024 Jan 9;3:122. Originally published 2023 Aug 4. [Version 2] doi: 10.12688/openreseurope.16142.2 (PMC10825415; doi:10.12688/openreseurope.16142.2)
Supplement: Peer review history

# Reviewer report: Reviewer response for version 2

**DOI:** 10.21956/openreseurope.18470.r37334

**Published:** 2024-01-29

**Authors:** Gavin Slade

**Affiliations:** 1 School of Sciences and Humanities, Nazarbayev University, Nur-Sultan, Kazakhstan

Thank you for your responses and for the edits to the paper. The edits meet all my points and I think the paper reads superbly in this revised form. The revised paper really does justice to the complexity of the social identities, structures, and contested power relations emerging in Russian prisoner societies.

 I really appreciate the extra care taken with the statistics, the pain-staking use of secondary sources, and the qualified claims about what we can say based on these sources, to set the piece up before going into the qualitative data.

 The interpretation of those qualitative data also reads really well now. In this revised version, you really convey the sense of the shifting dynamics and changing social distances among prisoners with diverse identities where who - or which groups - are more or less empowered is situational and not necessarily clear-cut.

 So, overall, this is an excellent and important contribution to the literature on an incredibly hard-to-access topic. I hope it will become a go-to study for scholars writing about prisoner society in Russia.

If applicable, is the statistical analysis and its interpretation appropriate?

Not applicable

Is the study design appropriate and is the work technically sound?

Partly

Is the work clearly and accurately presented and does it engage with the current literature?

Yes

Are the conclusions drawn adequately supported by the results?

Partly

Are sufficient details of methods and analysis provided to allow replication by others?

Yes

Are all the source data and materials underlying the results available?

No

Reviewer Expertise:

Prisons, crime and criminal justice in the former Soviet Union

I confirm that I have read this submission and believe that I have an appropriate level of expertise to confirm that it is of an acceptable scientific standard.

# Reviewer report: Reviewer response for version 2

**DOI:** 10.21956/openreseurope.18470.r37333

**Published:** 2024-01-22

**Authors:** Agnieszka Kubal

**Affiliations:** 1 School of Slavonic and East European Studies, University College London, London, England, UK

Excellent revision.

If applicable, is the statistical analysis and its interpretation appropriate?

Not applicable

Is the study design appropriate and is the work technically sound?

Yes

Is the work clearly and accurately presented and does it engage with the current literature?

Yes

Are the conclusions drawn adequately supported by the results?

Yes

Are sufficient details of methods and analysis provided to allow replication by others?

Not applicable

Are all the source data and materials underlying the results available?

Partly

Reviewer Expertise:

Socio-legal studies, qualitative methods, migration law in Russia and Eastern Europe, human rights

I confirm that I have read this submission and believe that I have an appropriate level of expertise to confirm that it is of an acceptable scientific standard.

# Reviewer report: Reviewer response for version 1

**DOI:** 10.21956/openreseurope.17426.r34282

**Published:** 2023-09-05

**Authors:** Gavin Slade

**Affiliations:** 1 School of Sciences and Humanities, Nazarbayev University, Nur-Sultan, Kazakhstan

This is a vitally important topic and it is great to see research of this type being undertaken to give us a better understanding of what is happening within Russian prisons during these times of opacity, closure and repression.

 The article is fascinating to read and makes a strong case for itself and the topic it covers. Yet, in my view, it requires a lot of work, particularly in the qualification of some of its claims. A process of streamlining and matching the data and methods with the scope and set up of the article is certainly required.

 I will try to elaborate on this latter overriding issue: 

 The paper sets itself up almost as a quasi-experiment: 

 It starts from the claim that there has been an 'explosion' of foreign national and particularly Muslim prisoners in Russia's prisons.

 It proceeds to the argument that: 

 This process has shifted the dynamics of Russian prison from condition X - one of 'penal cosmopolitanism' and/or apparent dominance by the criminal subculture - to condition Y: an ethnically and religiously fragmented social landscape. 

 This very bold hypothesis is stated here for example (the paper is provided in an online format so my apologies for no page numbers):

 'Our findings are that large-scale migratory processes post-1991 have transformed Russian penal institutions into a plural legal environment characterized by patterns of coexistence and conflict between the rules of formal and informal power nodes.'

 As it stands, the paper unfortunately does not present the appropriate data to statistically or logically infer that these are indeed the findings. 

 Starting with the initial premise - that there has been an explosion of foreign national and particularly Muslim prisoners - I hugely appreciate how hard it is to evidence this and I applaud the authors for a strong attempt to do so. But still, the question remained - to what extent is this true? The data the authors provide from 2015-2020 show a percentage point increase in foreign nationals in the prison estate in this period. This is not a huge increase and as the authors note in comparison to other jurisdictions in Europe it’s a quite small proportion of the prison population.

 Putting this issue to one side, since it could be accepted even anecdotally that there has been some influx of foreign nationals in the prison system, to what extent do the data presented demonstrate a change over time in the Russian prison system as a result of this explosion? The journey of Farhod through the system is fascinating but the narrative is of one person, in one prison colony in Russia, for a few years at only one time. I understand this was supposed to represent somehow the voices of 29 other respondents and there is absolutely nothing wrong with this as a method, however, it is not appropriate for building an argument around institutional *change over time* in the social order of overall Russian prisoner society. For this you would need, at the least, a pre- and post-event-of-interest-at-particular-time groups of respondents.

 Furthermore, the interpretation of the data was perplexing to me. It seemed to be trying to fit the data into the hypothesis the paper is pursuing rather than allowing the data to speak for itself.

 My neutral reading of Farhod's journey was very far from the idea that it somehow demonstrates the 'end of penal cosmopolitanism' (quote from the Conclusion). To the contrary, you describe a situation where a Chechen Muslim is informally in control of a penal colony. He variably empowers different groups of Muslims (and non-Muslims) depending on their attitude to the social order - presumably due to agreements with the prison administration and prisoner leaders in other prisons who are likely of other nationalities, at least we can assume they are not all Chechen. Certainly, the prisoners build networks of support based on zemlyachestvo and ethnic belonging. They also create parallel – but, for me, revealingly similar - informal institutions to the criminal subculture (korobka/obshchak). But I read this as telling us about adaptive strategies of identity formation within the bounds of a given social order. So, ultimately, on my reading religious and ethnic plurality is mixed together with criminal and subcultural identities in a way that does not indicate the *transformation* that the paper claims on a number of occasions. 

 In short, I was struck that this was not a depiction of an upended social order at all. Instead, this sounded like a story of the resilience of formal and informal institutions. This resilience involves the integration of emerging identities and accommodation of prisoner practices, belief systems and traditions. The data presented seemed to be a strong argument, as the authors say themselves, against the 'green wave' and 'green colony' idea, that traditional prisoner society was being 'overtaken' by Muslims that might turn radical, but it was also an argument against the idea that some 'new' – the authors use this term on a number of occasions - normative or legal pluralism has emerged. Instead, it seems a more boring – in a good way! - story about how social institutions evolve. 

 In support of this latter interpretation, I noticed that you did not much discuss instances of conflict which would really tell us if there were some new cracks or power lines being drawn. There are also a lot of *a priori* reasons to suppose that we would see evolution of older forms rather than a 'transformation' from some monolithic penal culture (which as you anyway note in conclusion probably – I would say definitely - never existed). Here are two pretty big reasons: one, the prison administration would not like to see such a transformation, and the prison administration is pretty powerful both informally and formally. Two, despite the, apparently, increasing numbers of Muslims and foreign nationals in the system the majority are still Slavic men, many of which will be non-Muslims, we don't hear much about them in the article due to the research design but theoretically we can assume an incentive to resist any 'transformation' that would disadvantage them. We can also assume they have an opportunity to act on their incentive (they are the largest group and have the closest social and symbolic distance to the majority of prison officers). Here's a nice study of how that works from Canada: Bucerius, S. M., Schultz, W., & Haggerty, K. D. (2023). “That shit doesn't fly”: Subcultural constraints on prison radicalization. *Criminology*, *61*(1), 157-181. Perhaps you do not describe any instance of conflict because the forms of solidarity you so fascinatingly describe (the jamaat, observance of Ramadan, cooking in separate spaces in the barrack) does not fundamentally redistribute resources or challenge existing hierarchies? 

 All of this is to say that I think the article needs to be qualified and rethought. It is an important article but its unit of analysis is the individual prisoner. That is, it is telling us something about migrant prisoner experiences, social positioning, code-switching and identity formation rather that a story of systemic change. Perhaps you can point to the more systemic element as part of this story but I am not sure with the data as is you can do more than that?

 Here are a few more concrete issues I had while reading that I think needs your attention (quotation marks indicate your text, my comment comes below in italics): 

 'The number of prisoners on remand in Moscow’s pre-trial detention facilities is vast, reflecting the high numbers of migrants working in the capital.'

*The sentence is missing something - probably the words 'foreign national' before 'prisoners'?*

 'Correctional colonies for convicted offenders in the oblasts of Moscow’s ‘penal catchment area’ can reach as much as 40% of the total number of prisoners in some institutions.'

*This doesn't make sense as a sentence as it stands.* 

 '300 radical prison jamoats'

*Should be 'jamaats' I guess, unless this is deliberately using a Tajik term in which case it should be explained.*

 'In theory, the moral panic surrounding prison jihad conflicts with the official prison service discourse portraying its facilities as sites of ethno-religious harmony (the 21st -century version of the ‘friendship of nations’ trope). It insists that the long tradition of ethnic neutrality in inter-prisoner and prisoner-administration relations remains intact and, in fact, constitutes a bulwark against the foreign-imported Islam in its prisons. It is principally the Tajik and Uzbek migrants, not Russia’s indigenous Muslims, who are understood to be vulnerable to the teaching of charismatic recruiters to the cause of violent Islam. This is attributed to their failure to integrate into mainstream prison society because of their poor command of Russian and cultural-outsider status. Isolated from other prisoners, compatriot ‘families’ ( *semeiki*) and larger groups of believers consisting of ‘petty criminals and drug dealers’ from Central Asia, who come together to pray, are believed to be easy prey for charismatic leaders of radical schools of Islam.'

*Could any of this be demonstrated? Perhaps through a quotation or at least source citation?*

 'The heirs to the *vory-v-zakony,'*

*Should read vory-v-zakone - you mention them here before you discuss and explain penal subculture so this could be confusing for the reader.*

 ‘Human rights advocates of penal reform use the term ‘neo-gulag’ to describe the current prison system in the Russian Federation.’

*Reference please – who used this term, I agree it is common but just would be good to cite someone.*

 ‘Prisoners in black colonies can indulge in card games ( *qimor*) at night and are expected to contribute to a mutual assistance fund ( *obshak*) administered by the sub-culture, and they are expected to take part in protests involving self-harm or hunger strikes, as directed by the sub-culture’s authority figures.’

*There is some mixing here between Uzbek and Russian words. Qimor is Uzbek I guess – so are the prisoners engaged in this only Uzbek? Would Russian prisoners also call it qimor?*

 As Russian penal space has had to accommodate the arrival of large numbers of transnational prisoners, new power hierarchies and social groups based on ethnic and religious lines have also emerged. Popularly, it is supposed that there are ‘green’ colonies and ‘green’ detachments or barracks to set alongside the ‘black’ and ‘red,’ in which Muslim prisoners dominate. In fact, the current policy in FSIN is to disperse Muslims among other prisoners unless they are convicted of serious terrorist offenses. In the latter case, the prisoner is confined in one of a small number of special regimes colonies or prisons that have cellular accommodation. Nevertheless, as we have already observed, the number of Muslim prisoners in some regions is such that they can form intermediate and higher-level social groupings or jamaats based on the tenets of Islam giving them a significant local presence in specific colonies. These groupings are led by an imam (religious leader) chosen by the prisoners because of their knowledge of Islam and Sharia law. These intermediate social groups are neither part of the traditional criminal subculture nor part of the administration-sanctioned hierarchy of activists. Instead, they position themselves as an autonomous community within prison society, membership of which is restricted to Muslims who commit to living and organizing their daily life according to Sharia Law. They invariably are ‘constructed’ as sub-cultural groupings by colony administrations, although they are not, as a matter of principle, necessarily oppositionist.

*Again this paragraph would read better with some demonstration or at least citation – where is this information coming from?*

 The example of this type of mutually supportive negotiation between prisoner leaders and the administration speaks more generally to the role that ethnic identity can play in the power geometry in Russian penitentiaries and, more specifically, in the case of black colonies, of the crucial role of the ethnicity of the leader of the sub-culture can play in protecting the interests of specific ethno-religious groups in Russian prisons today.

*In the text I had some discomfort about the ‘black’ and ‘red’ concepts since it is so disputed that there is any real distinction anymore and I guess that should be acknowledged. Here, and elsewhere, the text is written as if black colonies truly exist as a distinct category – maybe they do in Russia, but perhaps acknowledge the debate about this?*

 Examples of historical events that conferred status upon ethno-religious groups, included the 1920s Uzbek Basmachi uprising against Russian Imperial and Soviet rule which represents Uzbeks as fearless opponents of the state power. References to Tamerlane, founder of the Timurid Empire in the 14 ^th^ century, and now considered the founding father of the contemporary Uzbek state, was also a common reference point for Uzbek prisoners, further enhancing their reputation in prison as a fearless and rebellious nation.

*I really feel the data analysis could be more reflexive about what this is telling us – the claim to cultural capital is interesting but does not necessarily challenge in any serious way the social order.*

 Unlike the *korobka*, the thieves’ *obshak* is not compatible with Sharia law.

*This is presented as a challenge to the obshchak but I couldn’t understand what’s the difference and why korobka is Sharia-compatible but obshchak is not. Curious that they are both Russian terms too. Perhaps this should be commented on given that you are claiming that this korobka practice shows some ethnic distinction?*

 Furthermore, while latter-day Thieves-in-Law will insist that the *obshak* provides a safety net for less fortunate sub-culture members, in practice the first call on it is support for the comfortable lifestyle of the criminal leaders.

*Can it really be claimed that there is no abuse or corruption of the korobka system? I really doubt it – again, some reflexivity here about how your respondent might also be trying to present and idealize his ethnically and religiously bounded community, doesn’t mean it’s as pure and white as he is describing.*

 ‘the need to revise the prevailing understanding about the power dynamics in penal space in Russian penal institutions to take account of the ‘end of cosmopolitan penal space’ if such ever really existed in the USSR and recognize them today as sites of ethnic and religious pluralism.’

*The conclusion reveals the huge problem for an article like this when it is being set up as showing change over time – we just don’t know that those Muslim cultures weren’t present somehow in the past and what we see are adaptations and accommodations on previous formations rather than something, as you say at different points, ‘new’.*

*So, finally on this point, I would offer a word of caution: even if unintentional and even when you are clear you are not making an argument about radicalization, the assertion that there is a ‘new’ social reality and a redistribution of power towards Muslim subcultures this **might** play into the narrative of some sort of Muslim threat (we know when it comes to the Russian prison system that are a lot of malfeasant actors!). So I would go through the article and qualify, nuance and qualify again.*

If applicable, is the statistical analysis and its interpretation appropriate?

Not applicable

Is the study design appropriate and is the work technically sound?

Partly

Is the work clearly and accurately presented and does it engage with the current literature?

Yes

Are the conclusions drawn adequately supported by the results?

Partly

Are sufficient details of methods and analysis provided to allow replication by others?

Yes

Are all the source data and materials underlying the results available?

No

Reviewer Expertise:

Prisons, crime and criminal justice in the former Soviet Union

I confirm that I have read this submission and believe that I have an appropriate level of expertise to confirm that it is of an acceptable scientific standard, however I have significant reservations, as outlined above.

## References

[1] : “That shit doesn't fly”: Subcultural constraints on prison radicalization. *Criminology* .2023;61(1) : 10.1111/1745-9125.12327 157-181 10.1111/1745-9125.12327

# Response

**Published:** 2023-09-15

**Authors:** Judith Pallot

1. Thank you for your comments, we are grateful that you have taken the trouble to read our article carefully. You will see that we understand your criticisms and suggestions and will incorporate them - your observations have shone a spotlight on those places where we need to make our arguments clearer.  There are some places where we hope we can agree to disagree, but these are mainly where we are just coming at the problem you identify from a different angle from you.  
2. You are right that there are no statistical data to confirm the statement that there has been an ‘explosion’ of in-migrant prisoners and of central Asian origin in particular.  Our apologies for using language more fitting social media than in an academic article. However, there is a problem with your request to include supporting data; namely, that they simply do not exist in the public domain.  We have included the data (from Zemskov) for the Soviet period about Muslim prisoners, but these do not go beyond the end of the gulag. No data for the post-gulag period up to the present are available because of archive rules which keep them classified. Even Russian scholars working in the FSIN and Ministry of Justice Academies do not include statistics relating to the whole penal estate in their articles on “the migrant prisoner problem”.   For the period we are writing about in the article, we have a choice: either we assume there has been no change since 2000 in the proportion of the prisoner population made up by in-migrants from Central Asia, or we accept that, given the frequency of references in non-quantitative sources that refer to an increase in the size of the Muslim population in prisons, it is legitimate to conclude that something has been happening which deserves investigation and comment.  The reports of an increase in Muslim and Central Asian prisoners comes from a variety of different qualitative sources:  Russian-language scholarly articles, NGO reports, the popular press and broadcast media, and the interviews we have taken with former prisoners in Russian colonies (now in the several hundreds). We also were informed by Rustam’s and others’ work on labour migrants of Central Asian origin in Moscow and other cities, which reference the increase. We agree that these are anecdotal sources, and that we need to tone down the statement about there being an explosion in numbers and make clearer that we are interested in why, regardless of the numbers are, this has hit the headlines in recent decades.
3. However, we are confident in stating that there has been a substantial increase in foreign Muslim prisoners, which is what we are writing about in this article. Large numbers of prisoners classified as ‘foreign’ certainly is a new phenomenon that did not exist before 1991, simply because then central Asian migrants serving sentences were classified and treated as domestic, not as ‘foreigner’ prisoners. Prior to 1991, Russia did not have a foreign ‘migrant problem’ in its prisons. Indeed it needed only one prison for foreigners – in Leplei, Mordoviya – which was largely for drug traffickers and users from outside the USSR’s borders.   As far as FSIN is concerned, migrant prisoners are a new problem that it has to cope with.  We think it important to be sensitive to the danger of unconsciously arguing that people in the former republics can be understood as not much different from Russian citizens. We will reference the work on the foreign prisoner in western scholarship to underline the point that a comparator for the prisoners in our sample is foreign migrant prisoners in other jurisdictions.
4. Another point we should have made clearer that also speaks to the numbers question, is that foreign Central Asian migrants are clustered in specific remand prisons (SIZOs) and correctional colonies where they do constitute a disproportionately large share of the prisoner population.  In other words, we are writing about a correctional colony-UFSIN phenomenon, which is not necessarily nation-wide.  Our interviews have shown that there are oblasts where the prison population is more or less monocultural consisting exclusively of ethnic Slavs or Russians. But there are others which have a mix of several different ethnicities and yet others, where the majority population is Russian, but there is a large minority group that stands out.  It would be wonderful to have the data to be able to map all the variations, but 5 years of searching in the gulagechoes project hasn’t come across a source. We will make clear that when we are referring to the 21st century phenomenon of prisons having to cope with an influx of foreign Central Asian migrant prisoners, we are referring to a phenomenon that is geographically uneven.    
5. We strongly believe that the Uzbek and Tadjik nationals who migrate to Russia for work and end up in prison should not be equated with indigenous prisoners either in terms of how they are treated by the prison authorities or in terms of their experiences.  We may have misunderstood you, but the rejection of the exceptional nature of foreign prisoners of Central Asian origin seems to underpin your argument that prison society hasn’t been upended by their arrival in large numbers (in some places). The evidence we have is that the experience of foreign central Asian prisoners and their treatment is different from other ethnic minority prisoners who are Russian nationals. We have also interviewed Tatar Muslims and North Caucasus former prisoners and will be publishing articles about both these groups in the future. We can clarify our position on this issue and why it is important to our overall argument.
6. We will clarify what we mean by ‘penal cosmopolitanism’: when we write about penal cosmopolitanism, we are taking up the Soviet and post-Soviet claim that their prisons have always been ethnically neutral and have never discriminated on the basis of region, ethnicity/nationality or race.  There is no research on this topic in the late Soviet prison. The fact is that we are not in a position to say how typical, or representative is Farhod’s story. We can, however, say that it is not a story that we have encountered from archival, testimonies, and oral history interviews  for any period of Soviet penal history. We can make it clearer that we are not trying to generalise from Farhod’s story. 
7. On your specific comments:
  1. Conflict: we do not have examples of conflict. None of the interviews we took (with foreign in-migrant prisoners, and with the larger corpus of prisoners in general, refer to conflict between foreign in-migrant prisoners and other prisoners. Our interviews with prisoners serving sentences in the past twenty years almost universally paint a picture of harmonious ethnic relations (as do the gulag memoirs). Historically, ethnicity has been an issue in inter-prisoner conflict mainly in times of crisis; as it was in the 1950s during the Bitch wars, and the 1990s (when Chechens incarcerated during the Chechen wars being treated badly by officers).  We do not address Slavic men in the article because they are not our topic, here.  We can say, however, that the ethnic Russians and Russian nationals cannot be treated as an undifferentiated group. Our interviewees have described colonies as being criss-crossed with both horizontal and vertical social groupings,  which have a different degrees of power over the decisions affecting their daily lives and over the lives of others. (which, of course, are two different things)
  2. “Green colonies”: when Russian authors use the term green colony, they are signalling that they believe that the Muslims have ‘taken over’ the main sub-cultural position in colonies, displacing the ‘blacks’ / *Vory*.  We have not found evidence for this. Rather, the evidence that we have is that Muslims constitute a horizontal social grouping that has an intermediate status separate from the traditional sub-cultural hierarchical group(s) and the administration. In some colonies are sufficiently large to make a difference to how they are treated by other prisoners and the administration.  In this, we are agreeing with Elena Omel’chenko’s research about the emergence of intermediary social groups in colonies. Where we appear to disagree with you is whether such intermediate social groups do, or do not, challenge existing power hierarchies.  We do not believe that the in-migrant groups can be treated as simply another power hierarchy paralleling the criminal sub-culture and the *aktivisti*/administration.   The challenge they offer is, in fact, an alternative way of living their sentence. Lili di Puppo is writing about this is relation to north Caucasus prisoners.
  3. Resilience.  In the article, we are writing about changing geometry of relationships between social groups, not the resilience or not of pre-existing power nodes. No doubt the story about the Chechen watcher helps the resilience - the ability to adapt to changing circumstances – of the traditional sub-cultures in Russian prisons. Indeed, we suggest this as a possible motivation of the Chechen in question.  We can only guess at what the outcome will be of the emergence of Islamic jamaats in terms of the power in correctional colonies but we can already see that they have complicated the bi-polar dynamic of criminal sub-culture/administration in colonies.   Maybe the problem is in our use of the word “challenge.”  It is clear that prison administrations are trying to claw back the power they lost to criminal subcultures in the period 1980s-1990s+. In many places, they have managed to ‘re-colour’ colonies, in others there has been a partial shifts back to the administration, and yet others, there has been no change. In this article, we are giving an example of what can happen when sizeable numbers of foreign Central Asian migrants are inserted into a colony. 
  4. Following from the above, we want to stress that we believe that ethno-religious social groupings do represent a new phenomenon capable of upending the existing social order and power geometry in colonies. This is because they are ideologically distinct from both traditional sub-cultures and prison administrations.  You caution us to be careful about claiming novelty for the Muslim groupings because they may have existed in the past.  In fact, we know that religious observance was not permitted in the late soviet colony, and that the small groups of religious observers had to practice their religion secretly. The arrival of religion as a permissible in Russian prisons is absolutely new and, we believe, that when Muslim believers reach a critical number, they can change the power dynamic in prisoner society.  
  5. Black and Red. We agree with you here. We state in the article that we are employing the terms used by our respondents; i.e. the terms used by prisoners.  Red and black most probably don’t exist in any pure form (Oleinik made this observation on the basis of his interviews in the 2000s) and we know you are working on how to understand the relationship between authorities and sub-cultures in post-Soviet prisons at the present time. The evidence that we have from all our interviews is that whatever the theorizations penologists might have, as far as prisoners’ narratives are concerned, they frame their talk about prison in the black and red binary. We can clarify this and the consequences for our argument in the text.
  6. Where you have noted missing citations or a sentence isn’t clear we will make corrections.
  7. On Uzbek cultural capital. We include the discussion of this as, in our view, the shared history is grounds for the newly arrived  prisoner to choose to align with this group, rather than any other group in the prison. But we will have look at this to make what we mean to convey clearer.
  8. *Obshak*: the analysis that we have been doing with former prisoners from the north Caucasus confirms the horizontal nature of group formation among Muslims.  We think it is extremely problematic to assume that Muslim prisoners’ use of welfare funds mimic the use of funds in vertical criminal hierarchies.  In fact, our interviews with indigenous, as well as foreign Muslim former prisoners, also show that it works as genuine welfare, and it is consistent with Islam.  More generally, the evidence we have from our interviews is that social groups based on Islamic belief cannot be simply equated with the traditional hierarchically-constructed power groups in Russian colonies.  It is the basis of claims about the moral superiority of Muslim groups in prison.
  9. Radicalisation. If you read our comments as implying that “new” equals “threat”, then that is the opposite of what we are saying. We will make sure that we note that jamaat does not mean radicalisation toward terrorism.

# Reviewer report: Reviewer response for version 1

**DOI:** 10.21956/openreseurope.17426.r34279

**Published:** 2023-08-10

**Authors:** Agnieszka Kubal

**Affiliations:** 1 School of Slavonic and East European Studies, University College London, London, England, UK

The article titled "Ethnic and Religious Identities in Russian Penal Institutions: A Case Study of Uzbek Transnational Muslim Prisoners" addresses the evolving landscape of Russian penal institutions as a result of significant migration patterns and their impact on ethnic and religious diversity within the prison system. The study's contribution to scholarship lies in its exploration of how large-scale migration to Russia from Central Asian countries has transformed the demographic composition of Russian prisons and challenged prevailing assumptions about the neutrality of the penal system.

 The methodology employed involves person-to-person interviews conducted in Uzbekistan with individuals who have served sentences in Russian penal institutions over the past two decades. This qualitative approach offers valuable first-hand perspectives on the experiences of transnational Muslim prisoners. The study effectively highlights the complex interplay between formal prison regulations, informal subcultures like the "thieves' law" and ethnic solidarity norms, and religious influences such as Sharia law. This methodological approach enhances the richness of the data, allowing for a nuanced understanding of the dynamics within the prisons.

 The quality of data analysis is evident in the article's exploration of how crime, interpersonal conflicts, and fabricated charges contribute to the diverse prisoner population. The authors demonstrate how ethnic and religious identities intersect within the prison environment, leading to clashes and coexistence of different norms and systems of authority.

 The conclusions drawn from the study are adequately supported by the results. The article challenges the notion of Russian penal spaces as ethnically neutral and cosmopolitan by revealing how the influx of transnational prisoners has introduced a new dimension of ethnic and religious pluralism. By showing that the prison environment is shaped by a range of factors beyond the official rhetoric, the study disrupts prevailing assumptions and calls for a re-evaluation of power dynamics within Russian prisons.

 In summary, the article significantly contributes to scholarship by shedding light on the transformation of Russian penal institutions due to migration patterns. Its methodology, centered on qualitative interviews, provides valuable insights into the experiences of Uzbek transnational Muslim prisoners. The data analysis is comprehensive, effectively presenting the coexistence and clashes of different norms within the prison context. The conclusions drawn are well-supported by the research findings, challenging prevailing notions and offering a more nuanced understanding of the ethnic and religious dynamics within Russian prisons.

 It would be my personal interest to explore in further research the role of human rights law during Russia's membership of the Council of Europe and its recognition of the jurisdiction of the European Court of Human rights as shaping the varied and multidimensional experiences of the multicultural prison.

If applicable, is the statistical analysis and its interpretation appropriate?

Not applicable

Is the study design appropriate and is the work technically sound?

Yes

Is the work clearly and accurately presented and does it engage with the current literature?

Yes

Are the conclusions drawn adequately supported by the results?

Yes

Are sufficient details of methods and analysis provided to allow replication by others?

Not applicable

Are all the source data and materials underlying the results available?

Partly

Reviewer Expertise:

Socio-legal studies, qualitative methods, migration law in Russia and Eastern Europe, human rights

I confirm that I have read this submission and believe that I have an appropriate level of expertise to confirm that it is of an acceptable scientific standard.

# Response

**Published:** 2023-08-11

**Authors:** Rustamjon Urinboyev

**Affiliations:** Sociology of Law, Lunds Universitet, Lund, Skåne County, Sweden

Dear Agnieszka, Thank you so much for your review. We will consider your suggestion regarding the human rights aspect in our future work.  Best regards, Rustam

# Response

**Published:** 2023-09-15

**Authors:** Judith Pallot

Thank you for your comments Agnieska and my apologies for not responding to you queries sooner.  

 The question of how prisoners of different origins understand and access knowledge about human rights is very important. Have you seen Laura Piacentini’s book “The virtual reality of imprisonment in Russia: “preparing myself for prison in a contested human rights landscape”?

 This discusses how rights consciousness is affected by the (illegal) use of modern communication technologies and social network sites by prisoners in Russia.  As a general rule, in Russian penal institutions, rights are understood narrowly as the ‘right to complain’ and there are numerous channels prisoners can use to complain, most leading nowhere because the supposedly independent monitors have been subject to regulatory capture.  In various extradition cases I have prepared reports for, I have been struck in the responses from the general prosecutors’ office about rights compliance in prisons also by how rights are reduced to the physical conditions of detention m ^2 ^per head of space, ventilation and so on., what Laura refers to as “norm compliance”.   Of course, Russia harmonised its legislation on prisoners’ rights with the Council of Europe, but the issue is its implementation.

 There is a report about the treatment of Chechen prisoners that Svetlana Gannushkina drew our attention to that list the violations of Muslim’s rights and have been appealed to the European Court https://refugee.ru/wp-content/uploads/2015/09/prison-report_eng.pdf   The local courts in Russia were getting better before Russia was evicted from the COE hearing complaints/appeals from prisoners about conditions of detention, but usually they simply pay compensation, rather than requiring changes in the prison service. Foreign migrants are at a disadvantage because the notices hanging in prison are in Russian, the low quality of advocates they are allocated and prioritization of domestic Muslims by NGOs. The NGOs we have investigated have been increasingly reluctant to take up ethic minority cases and choose carefully which cases to publicise through the media. Migrant prisoners are sent back to Uzbekistan and Tajikistan as soon as their sentence is finished and, according to some we interviewed the receiving authorities are given a report on whether they were ‘converted’ while in prison and prayed and attended the prayer room regularly.      

 One of my post-docs chased down case law on ethnicity and religion in the ECtHR. The cases that have gone to court come from all over the former SU and Eastern Europe. Most of the cases are Article 3 - torture, inhuman and degrading treatments and punishments, Article 14 – protection from discrimination. I have been surprised by some of the judgments which have run counter to what I would, as a layperson, have expected; for example, the Court accepted the argument that the prison service did not have to provide kosher food for an orthodox Jewish prisoner  if relatives  lived nearby and could supply it from outside. This is relevant to migrant labourers who are usually connected to the Uzbek and Tajik communities that provide them with food, even though their families are thousands of miles away. But I haven’t seen a case on Halal from a migrant  in a Russian colony; this doesn’t mean there have been an applications on this.   

 I was also surprised by another ruling that it is not discriminatory to prevent a Muslim from praying five times a day because there are other ways of exercising beliefs in prison.  I have been assured if I were a lawyer, I wouldn’t be surprised at these rulings! It is all apparently a product of the margin of appreciation.  

 I am sorry I cannot be of more help.
